# Genetic Determinants of Phosphate Response in *Drosophila*

**DOI:** 10.1371/journal.pone.0056753

**Published:** 2013-03-08

**Authors:** Clemens Bergwitz, Mark J. Wee, Sumi Sinha, Joanne Huang, Charles DeRobertis, Lawrence B. Mensah, Jonathan Cohen, Adam Friedman, Meghana Kulkarni, Yanhui Hu, Arunachalam Vinayagam, Michael Schnall-Levin, Bonnie Berger, Lizabeth A. Perkins, Stephanie E. Mohr, Norbert Perrimon

**Affiliations:** 1 Endocrine Unit, Massachusetts General Hospital, Boston, Massachusetts, United States of America; 2 Dept. of Genetics, Harvard Medical School/Howard Hughes Medical Institute, Boston, Massachusetts, United States of America; 3 Mathematics Department and Computer Science and Artificial Intelligence Lab, MIT, Cambridge, Massachusetts, United States of America; Queen Mary University of London, United Kingdom

## Abstract

Phosphate is required for many important cellular processes and having too little phosphate or too much can cause disease and reduce life span in humans. However, the mechanisms underlying homeostatic control of extracellular phosphate levels and cellular effects of phosphate are poorly understood. Here, we establish *Drosophila melanogaster* as a model system for the study of phosphate effects. We found that *Drosophila* larval development depends on the availability of phosphate in the medium. Conversely, life span is reduced when adult flies are cultured on high phosphate medium or when hemolymph phosphate is increased in flies with impaired Malpighian tubules. In addition, RNAi-mediated inhibition of MAPK-signaling by knockdown of *Ras85D, phl/D-Raf* or *Dsor1/MEK* affects larval development, adult life span and hemolymph phosphate, suggesting that some *in vivo* effects involve activation of this signaling pathway by phosphate. To identify novel genetic determinants of phosphate responses, we used *Drosophila* hemocyte-like cultured cells (S2R+) to perform a genome-wide RNAi screen using MAPK activation as the readout. We identified a number of candidate genes potentially important for the cellular response to phosphate. Evaluation of 51 genes in live flies revealed some that affect larval development, adult life span and hemolymph phosphate levels.

## Introduction

Inorganic phosphate, the mono- or divalent anion of phosphoric acid [HPO_4_^3−^, H_2_PO_4_^2−^], is required for cellular functions such as DNA and membrane lipid synthesis, generation of high-energy phosphate esters, and intracellular signaling. Cells and organisms have developed elaborate mechanisms to assure an adequate supply of phosphate. Yeast is the only eukaryote, however, in which the genetics of cellular phosphate homeostasis is understood [1]. When yeast cells are starved of phosphate, the phosphate-dependent cyclin-dependent-kinase (CDK) inhibitor *Pho81* inactivates the *Pho80/Pho85* cyclin/CDK complex. As a consequence the unphosphorylated basic helix-loop-helix transcription factor *Pho4* associates with the nuclear import receptor *Pse1* to enter the nucleus and binds to a phosphate response element (PRE) [2] in genes belonging to the yeast Pho-regulon that permit the cell to better assimilate phosphate from the surroundings [3], [4]. The Pho-regulon includes genes coding for high affinity phosphate transporters (*Pho84, Pho89*) and secreted acid phosphatases (*Pho5, Pho11, Pho12*) [3].

Compared to what is known for bacteria and yeast, little is known about the metabolic effects of phosphate in metazoan species [5], [6]. Recent evidence suggests that the mammalian ortholog of yeast *Pho89, Pit1*, mediates cellular effects of phosphate [7], which can be blocked by the addition of phosphonoformic acid (PFA), a competitive antagonist of phosphate transporters and cellular phosphate uptake [8], [9]. In addition, we recently showed that orthologs of yeast *Pho84* mediate activation of MAPK in *Drosophila* cell lines [10]. Thus, multiple membrane transporter families may be involved in cellular phosphate uptake and intracellular phosphate may be what is sensed in metazoan species. Activation of MAPK by inorganic phosphate at physiological concentrations between 5–10 mM was also demonstrated in multiple mammalian cell lines [7], [11], [12], [13], [14] and may be required for osteoblastic and chondrogenic differentiation, or pathological osteogenic differentiation of vascular smooth muscle cells in response to phosphate [5], [6].

In multicellular organisms phosphate needs to be absorbed from the diet in the gut to enter the circulation and to be available for cells. Circulating phosphate levels, total body phosphate content and excretion by the kidneys are tightly regulated by a number of hormones. In mammals these hormones are *fibroblast growth factor 23 (FGF23), parathyroid hormone (PTH),* and *1,25-dihydroxy vitamin D (1,25(OH)_2_D)* [6], [15] and genetic or acquired disturbances of these regulatory mechanisms can result in serious human disorders [15]. Serum phosphate feeds back to regulate these factors in a homeostatic fashion [15], with high phosphate increasing the secretion of *PTH* and *FGF23* and low phosphate stimulating the synthesis of *1,25(OH)_2_D*, the active form of vitamin D [6]. However, it remains unclear, whether activation of MAPK by phosphate has a role in the homeostatic regulation of *FGF23, PTH or 1,25(OH)_2_D* at the level of gene expression [16].

The clinical consequences of severe hypophosphatemia (too little phosphate), which for example are seen in conditions of malnutrition or tumor-induced hypophosphatemia [17], include hemolysis, skeletal muscle myopathy, cardiomyopathy, neuropathy, and osteomalacia and, in some cases, contribute to death. On the other hand, hyperphosphatemia (too much phosphate) due to familial tumoral calcinosis [18] or chronic kidney disease (CKD) leads to tissue calcifications [19] and metabolic changes, which to date are poorly understood. Outcomes in patients with CKD and hyperphosphatemic mouse models can be improved by dietary phosphate restriction or treatment with phosphate binders such as sevelamer (Renagel), which reduce phosphate absorption from the diet and normalize circulating phosphate levels [20], [21]. Thus, gaining an understanding of the specific pathways and mediators that regulate cellular and extracellular phosphate levels might lead to the development of improved dietary or therapeutic approaches to the treatment of both hypo- and hyperphosphatemia.

Here, we show that *Drosophila melanogaster* can be used as a model organism to design genetic screens for metabolic and homeostatic phosphate effects and describe the effects of *in vivo* RNAi-mediated knockdown of 51 modifiers of phosphate-induced MAPK in S2R+ cells on larval development, adult hemolymph phosphate and life span.

## Methods

### Fly Stocks and Culture

Fly stocks were cultured as described in Methods S1 on standard medium (SM) or defined medium (DM) [22] to which 30 mM sodium-phosphate (pH 6.0), 30 mM sodium-sulfate (pH 6.0), 0.1–10 mM phosphonoformic acid (Sigma P6801), or 0.1–3% sevelamer (gift from Dr. Yves Sabbagh, Genzyme, Inc., [23]) were added. Sevelamer is an FDA-approved drug that is used in patients with CKD to reduce absorption of dietary phosphate. The soluble fraction of phosphate in SM is about 3.7 mg/dl (1.2 mM), when measured as ammonium-molybdate adduct using the Liqui-Phospho UV reagent (Standbio Laboratory, TX). Supplementation of this medium with 1% sevelamer reduced the fraction of soluble inorganic phosphate to 0.16 mg/dl (0.05 mM). Supplementation with 30 mM sodium phosphate (pH 6.0) raised the soluble inorganic phosphate to 110 mg/dl (35 mM). As changes in osmolarity, caused by the addition of increasing amounts of phosphate, might lead to defects that are not specific to phosphate, but for example due to osmolar stress, we performed tests with sulfate alongside with phosphate. Finally, dye-feeding assays [24] were used to confirm similar consumption of the different foods.

### Larval Development, Life Span and Hemolymph Phosphate Assays

To evaluate wild-type larval development 20 mated females were allowed to lay approximately 100 eggs for 2 days. Adults were discarded and culture was continued at 18°C, 25°C, or at 29°C. Near the middle of the L3 stage, vials were inspected daily to determine the number of animals at different developmental stages (*i.e.* climbing L3 larva, prepupa, pupa, adult fly male/female).

Life span was evaluated by culturing 30–40 young adults separated by gender on SM or DM with different supplements as described in Methods S1. In initial tests comparing males and females, we were able to rule out gender-specific effects of dietary phosphate supplementation or RNAi-knockdown. Thus, we subsequently conducted life span tests using males.

To collect excretions sibling females fed different media at 18°C, 25°C or 29°C for 5 days were transferred to SM for 60 min., followed by a 60 min. collection of excretions in a 1.5 ml vial. For hemoymph collections, female flies were anesthetized with CO_2_, heads were removed, and fly bodies were centrifugated at 5000 rpm for 3 min. at 4°C in a 200 ul eppendorf vial with a punctured bottom, allowing for the collection of the clear, cell-free hemolymph. For whole fly phosphate bodies were homogenized in assay reagent. The excretion, hemolymph and whole fly samples were then cleared by centrifugation and assayed for phosphate concentration in 100 ul ammonium molybdate phosphate assay reagent (Phospho Liqui-UV, Stanbio 0851-250) at 340 nm.

### Genome-wide *Drosophila* Cell-based RNAi Screen (Primary Screen)

The genome-wide dsRNA screening collection of the *Drosophila* RNAi Screening Center (DRSC) covering >95% of the *Drosophila* genome has been described previously [25]. Approximately 14,000 genes were screened in S2R+ cells [26], using an in-cell immunohistochemical assay for dual-phosphorylated ERK1/2 (Cell Signaling Technologies, Inc #CS4370), DAPI as counter stain to correct for cell number, and 10 mM sodium phosphate as the stimulus along with control as described in Methods S1 and Figure S6A–C. The primary list of “hits” (positive results) comprised 1924 genes, including 5 genes of the canonical MAPK pathway (*i.e. downstream of receptor kinase (drk), downstream of raf1 (Dsor1), ras85D, sos, corkscrew*) and 11 genes that are known inputs or effectors of MAPK (*i.e. slipper, mushroom bodies tiny, protein tyrosine phosphatase-ERK/enhancer of ras1, nemo, eiger, RH51659P, SD03870P, CG32703-PA, CG14217-PD, sevenless*, *pointed*). We excluded hits not expressed in S2R+ cells and annotated the remaining genes for molecular function, conservation, and other features by cross-referencing with publicly-available databases i.e. DAVID [27], fly-MINE [28] and by manually annotating the list. Our goal was to retain genes that encode conserved proteins with known or putative membrane-spanning molecules, as well as known or putative receptors, kinases, phosphatases and transcription factors, but to exclude genes required for routine basic cellular functions, genes for which we could not detect known or putative human orthologs, or genes previously identified as important for insulin-mediated activation of MAPK [29]. A subset of 555 genes was selected for validation using at least two independent dsRNA amplicons per gene as described in the results section and Methods S1 using 10 mM sodium phosphate buffer or with 50 ug/ml human insulin as stimulus.

### *In vivo Drosophila* RNAi Screen (Secondary Screen)

At least two independent transgenic RNAi-lines targeting 51 of the 146 final hits were available (TRiP, http://www.flyrnai.org/TRiP-HOME.html, VDRC, http://stockcenter.vdrc.at/control/main) and crossed with temperature-regulated ubiquitous *Gal80*/*Gal4* driver stocks using the *daughterless* (*da*) promotor to drive *Gal4* expression (*w-, hs-hid(y)/w-;tub-Gal80^ts20^;da-Gal4*; referred to as *da-Gal4^ts^*), and confirmed using the *alphaTub84B* promotor (*w-*;*tub*-*Gal80* ^ts10^;*tub-Gal4/TM6B*; referred to as *tub-Gal4^ts^*)) [30].

*da-Gal4^ts^>*UAS-RNAi animals were examined for larval lethality at 29°C, the inducing temperature. Adult males were reared at 18°C to keep the RNAi un-induced during development and used within three days of eclosing for life span assays. Sibling females were used for hemolymph phosphate assays as described above. Life span of F1 males generated with control RNAi-lines targeting the *white* gene (TRiP# HMS00017, VDRC #30034) at 29°C on SM was 31+/−1 days, and 35+/−0.4 days, respectively, targeting green fluorescence protein (*GFP)* (Bloomington ID # 35785) was 35+/−1.4 days, and targeting *luciferase (Luc)* (TRiP # JF01355) was 39+/−2 days. Hemolymph phosphate of F1 generation females targeting *white* (TRiP #HMS00017, VDRC #30034) was 25+/−2.5 mg/dl and 31+/−1.7 mg/dl, respectively, targeting *GFP* (Bloomington ID # 35785) was 27+/−2 mg/dl, and targeting *Luc* (TRiP # JF01355) was 29+/−2.4 mg/dl.

Off-target effects were unlikely, if results were reproducible by at least two independent RNAi-lines and significant based on p<0.05 (Student’s t-test) across all RNAi-lines targeting a single gene when compared to control hairpins targeting *white, GFP* and *Luc*. Genetic background effects were unlikely since phenotypes were generally absent in the uninduced state (18°C) (see Figs. S1A, S9).

### Data Analysis

Median life span and maximum life span were calculated for each vial of flies using Prism 5.0 d (GraphPad Software, CA), averaged between multiple vials based on a total of 120–240 flies of each genotype and medium. Student’s T-test (two-sided, unequal variance) was then used to determine significant differences between genetic mutants and treatment groups (p<0.05). To correct for multiple comparisons we applied Bonferroni’s method [31] to re-test outliers. Hierarchical clustering was performed in Cluster 3.0 [32] using Pearson correlation or Euclidean distance, and displayed using Java TreeView 1.1.6 [33]. Final hits were annotated with GO term categories using the DAVID tool (http://david.abcc.ncifcrf.gov/) [27], and FlyMine (www.flymine.org/) [28]. Candidate human and murine orthologs were obtained using DIOPT (http://www.flyrnai.org/cgi-bin/DRSC_orthologs.pl) [34] and annotated with murine knockout or over-expression phenotypes at MGI if available (http://www.informatics.jax.org/) [35], and with human disease phenotypes using DIOPT-DIST (http://www.flyrnai.org/cgi-bin/DRSC_DG_query.pl) [34].

## Results

### Phosphate is Required for *Drosophila* Larval Development

**Figure 1:**
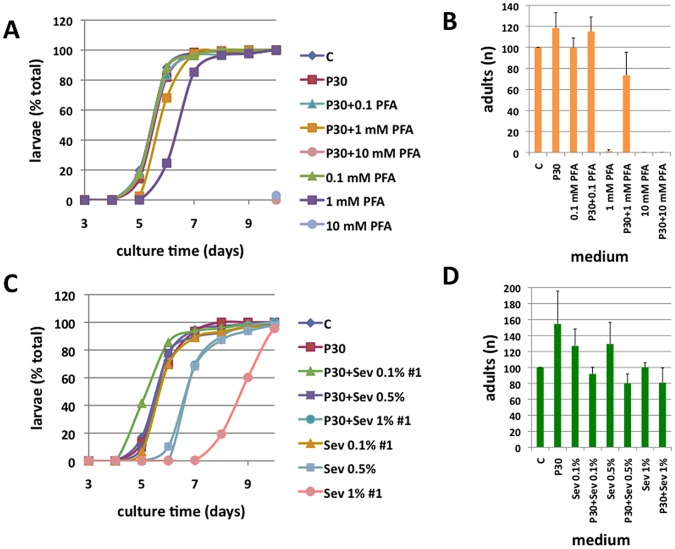
The effect of sevelamer and PFA on larval development is reversible by addition of 30 mM phosphate to the medium. **A, C:** Number of larvae emerged from the medium. P30 = standard medium supplemented with 30 mM sodium phosphate (pH 6.0), PFA = phosphonoformic acid 0.1–10 mM, Sev = sevelamer 0.1–1%; Shown are means, CV<10%, **B, D:** Number of adults eclosed. Shown are means ± SEM of one representative experiment, performed in triplicate.

Culture of three commonly used laboratory fly strains, *y w*, Canton S (CS) and Oregon R (OR), on standard medium (SM) or SM supplemented with 30 mM sodium phosphate (P30) equally supported larval development and eclosion of adult flies after 10 days at 25°C. To investigate the effects of phosphate on larval development, we added phosphonoformic acid (PFA) to block sodium-phosphate co-transporters and cellular uptake of phosphate [36]. Supplementation of SM with 0.1 mM PFA did not result in developmental delay or lethality of *y w* flies. However, developmental delay was noticeable in *y w* animals fed SM with 1 mM PFA and led to pupal lethality. Larvae reared on SM with 10 mM PFA died at the first instar stage unless the medium was further supplemented with 30 mM sodium phosphate (Fig. 1A and 1B). Thus, we observed a dose-dependent response to PFA treatment that could be reversed by supplementation with dietary phosphate. To confirm phosphate-dependence of larval development we used the anion exchange resin sevelamer to inhibit dietary uptake of phosphate [23]. When *y w* larvae were reared on SM supplemented with 0.1% sevelamer, no effect on larval development was observed. However, increasing the dose to 0.5% delayed emergence of L3 larvae from the medium, puparation and eclosion of adults by at least one day (Fig. 1C, Fig. S2). Similar to what we found for PFA, the effect of sevelamer could be rescued by supplementation with 30 mM sodium phosphate. A dose of 1% sevelamer (Sev1%) resulted in a more severe developmental delay that could be prevented by the addition of 30 mM sodium phosphate. Similar results were obtained with the two wildtype strains, CS and OR (Fig. S2), and males and females eclosed at similar ratio after development on foods with different phosphate contents.

### Dietary Phosphate Affects Life Span of Adult Flies

**Figure 2:**
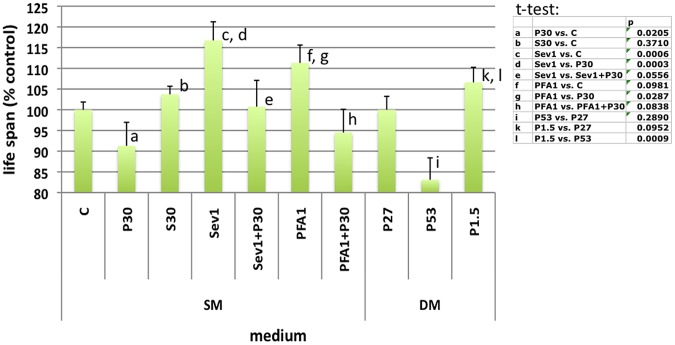
Phosphate supplementation or inhibitors of phosphate uptake influences adult life span. Median life span of adult *y w* males cultured on standard medium (SM): Control (C, n = 550), 30 mM sodium phosphate (P30, n = 465), 30 mM sodium sulfate (S30, n = 245), 1% sevelamer (Sev1, n = 207), 1 mM phosphonoformic acid (PFA1, n = 130), and the combinations: Sev1+P30 (n = 115), PFA1+P30 (n = 137), or defined medium (DM) supplemented with 1.5, 27, or 53 mM sodium phosphate (P1.5, n = 202; P27, n = 200; P53, n = 194). P<0.005 was used to test for multiple comparisons between eleven treatments.

Dietary phosphate modifies circulating phosphate levels and life span in genetically modified mice [21], [37], [38] and humans with CKD [19], [20], [39]. We next reared flies on SM and then placed F1 adults within 3 days of eclosing on experimental media to ask if dietary phosphate could modify adult life span. The median adult life span of *y w* males at 25°C was 42±0.8, 38±2.4 (p = 0.02 vs. SM), and 44±0.8 (not significant vs. SM) days when adults were cultured on SM, P30, or 30 mM sodium sulfate (S30), respectively (Fig. 2). Findings were similar for females (51.2±0.5, 39.2±2.6 (p = 0.04 vs. SM), and 46.8±0.2 (p = 0.01 vs. SM) on SM, P30, and S30, respectively) and for CS and OR males (Fig. S3A). The effects of phosphate were dose-dependent between 15 and 60 mM (Fig. S3B).

If increasing phosphate levels through dietary availability results in shortened life span, then conversely, reducing phosphate intake might extend life span. Consistent with this idea Sev1% increased median life span to 49±1.9 days (p = 0.006 vs. SM and p = 0.003 vs. P30), an effect that was lost when we additionally supplemented with 30 mM sodium phosphate. Similarly, adult life span was extended to 47±1.8 days (p = 0.03 vs. P30) when 1 mM PFA was added to SM but not when adults were cultured on SM with both 1 mM PFA and 30 mM sodium phosphate. Neither phosphate, nor sevelamer affected the rate of food consumption (Fig. S4A).

To further establish that the effect of sevelamer and PFA on improving life span is not due to non-specific effects, we cultured flies on Robert’s defined medium DM [22]. Although overall life span was reduced on this medium, presumably as it lacks components present in the more complex SM, we observed statistically relevant differences when *y w* males, reared on SM, were cultured on DM supplemented with phosphate for the remainder of their life span. Specifically, we observed a significant inverse relationship between life span and phosphate concentrations (Fig. 2).

**Figure 3:**
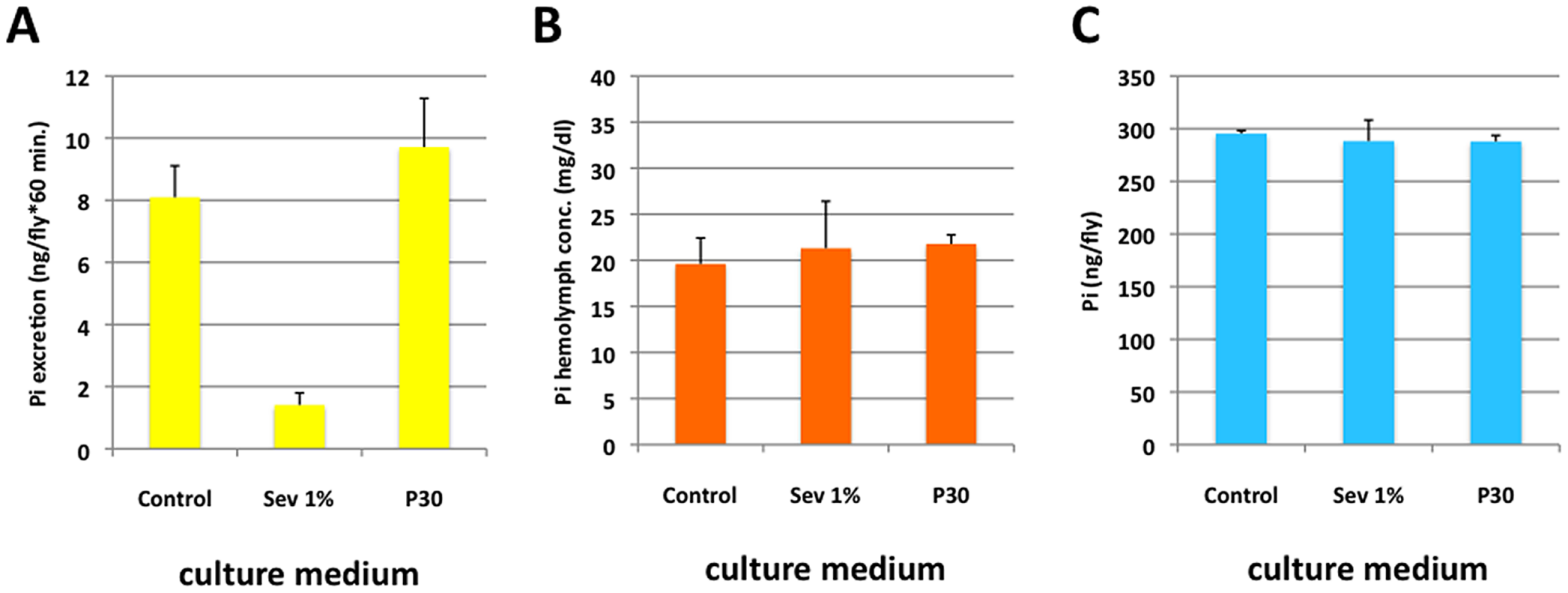
Adult hemolymph phosphate, phosphate excretion and whole fly Pi. A: Excretion of phosphate after culture of *y w* females for five days on standard medium (C) alone or supplemented with 1% sevelamer (Sev1%) and 30 mM sodium phosphate (P30) (n = 3 pooled collections of 15–20 flies). **B:** hemolymph phosphate concentration (n = 3 pooled collections of 15 flies) and **C:** whole fly phosphate (n = 10) of flies cultured as described for A. Note that *y w* and CS flies have lower hemolymph phosphate concentrations than OR and *w^1118^* flies and the F1 generation females that express control RNAis (see Methods and Fig. S5), which is likely due to differences in genetic background.

Since dietary phosphate levels influence life span, we asked whether this effect is mediated by a change in hemolymph phosphate levels, which in turn may cause extracellular mineralization as seen in higher species or cellular toxicity [21], [37], [38], [40]. When *y w* females are cultured on SM, Sev1%, and P30 for five days, excretions directly reflect intake of phosphate (Fig. 3A). However, the hemolymph phosphate concentration and whole fly phosphate content of these flies were indistinguishable across all culture conditions (Fig. 3B and 3C). Similar results were obtained for *w^1118^*, CS and OR strains (Fig. S5). The ratio of phenol red over FD&C blue1, food dyes [24] that measure urine and fecal dye-excretion or fecal dye-excretion alone, respectively, indicated that excess phosphate may simply travel through the gut and be excreted in feces, and/or actively excreted into the gut following uptake (Fig. S4C). The stability of hemolymph phosphate in the face of wide variations in exposure of the organism to dietary phosphate suggests strong homeostatic mechanisms maintaining that stability.

### Adult Hemolymph Phosphate is Dependent on the Function of the Malpighian Tubules

**Figure 4:**
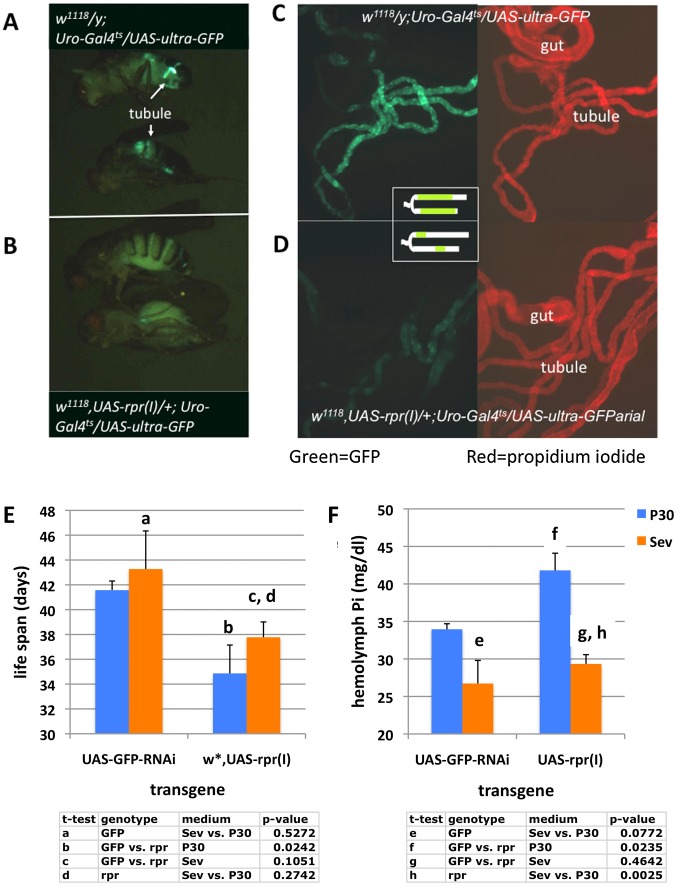
Principal cell ablation increases hemolymph phosphate and decreases life span, rescue by addition of sevelamer to the culture medium. A, B: Micrographs of 10 days old males with expression of GFP (green) in principal cells (**A**) or females with expression of GFP along with *reaper* (*rpr*) in principal cells after culture for ten days at 29°C (**B**). Epifluorescent microphotograph (10X) of tubule and gut of the same genotypes shown in A+B. Ablation of GFP-positive cells (green) by *rpr* is nearly complete with the exception of a few segments as shown schematically in the inset. As a consequence when compared to *rpr*-negative tubules (**C**), the majority of the tubule stained with propidium iodide (red) is made up of GFP-negative cells in (**D**). Median life span (**E**, n = 60–120 per condition) or hemolymph phosphate concentration (**F**, n = 3 with collections from 15 flies) of females expressing *rpr* or *GFP*-RNAi in principal cells after culture on standard medium containing 30 mM sodium phosphate (P30) normalized when cultured on 1% sevelamer (Sev1%) for 14 days.

The kidneys in higher species are responsible for excretion of phosphate in the setting of high dietary load and re-absorption of phosphate from the urine when dietary supply of phosphate is low. Malpighian tubules are the phylogenetic ancestor of the renal tubes [41]. They are composed of two cell types: stellate cells, which are important for water and chloride excretion, and principal cells, which are important for the excretion of cations and organic solutes. To determine the role of principal cells in phosphate homeostasis, we expressed the pro-apoptotic gene *reaper* (*rpr*) [42] using a *urate-oxidase* (*Uro)-Gal4;tub-Gal80^ts7^* driver (*Uro-Gal4^ts^*), which permits gene expression in a temperature-inducible fashion in principal cells. At the inducing temperature, epifluorescent and confocal analyses confirmed the near-total ablation of GFP-positive cells by *rpr* when coexpressed in principal cells (Fig. 4A, 4B). As a consequence, when compared to *rpr*-negative flies (Fig. 4C), the majority of the tubule in *rpr*-positive flies is made up of *urate-oxidase* negative cells in Figure 4D. Thereby impaired function of principal cells resulted in a female life span of 34±2.2 days on P30 medium (vs. 42±0.9 days for control flies, p = 0.024). Hemolymph phosphate in these flies was raised to 42±2.3 mg/dl (vs. 33±0.7 mg/dl for control flies, p = 0.023) (Fig. 4F). Importantly, this effect could be overcome by the addition of 1% sevelamer to the medium, resulting in lower hemolymph levels and improved life spans (Fig. 4E). Taken together these findings suggest that dietary phosphate increases hemolymph phosphate, which inversely correlates with longevity, at least in the setting of tubule failure. These results parallel what is observed in human patients with CDK, who similarly have increased circulating phosphate concentrations, and for whom blocking phosphate-uptake from the diet with sevelamer can improve outcomes.

### RNAi-mediated Inhibition of MAPK-signaling in vivo Decreases Hemolymph Phosphate

**Figure 5:**
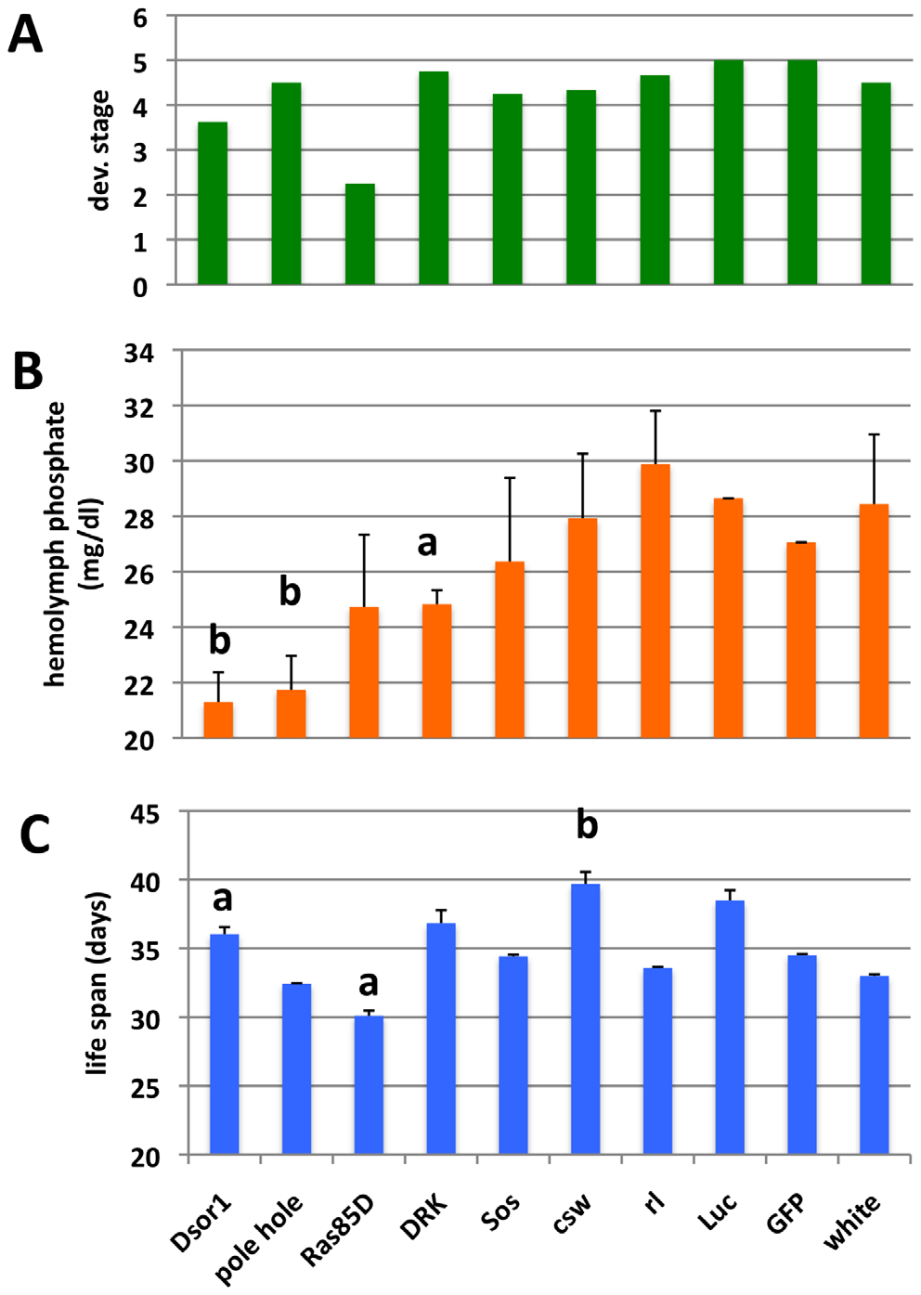
RNAi-mediated inhibition of MAPK-signaling *in vivo* decreases hemolymph phosphate. A: The most advanced developmental stage with induced knockdown was scored on standard medium (1 = embryonic, 2 = first and second instar larva, 3 = third instar larva, 4 = pupal lethal, 4.5 = developmental delay, adult, 5 = adult). **B:** hemolymph phosphate concentration of young adult females cultured at 29°C for five days, and **C:** median life span of adult males cultured at 29°C on standard medium (see also Table S1). **a:** p<0.05, **b:** p<0.007 vs. Luc/GFP/white controls. P<0.017 was used to test for multiple comparisons between three treatments.

Previously, we showed that activation of MAPK by phosphate is evolutionary conserved in *Drosophila* S2R+ hemocyte-like cultured cells and likely requires the function of major facilitator superfamily (MFS) sodium-phosphate co-transporters [10] (Fig. S7). When we decreased MAPK-signaling activity *in vivo* by RNAi-mediated knockdown of *drk/GRB2, Ras85D, phl/D-Raf and Dsor1/MEK* using *da-Gal4^ts^* for a relatively short period of five days, hemolymph phosphate was decreased in adult flies (Fig. 5B). Likewise, knockdown of *corksrew (csw/SHP2),* a phosphatase known to stimulate RTK input into the MAPK pathway [43], and *Sos* mildly reduced adult hemolymph phosphate. Therefore, hemolymph phosphate is genetically downstream of MAPK signaling. Knockdown of these genes also impaired larval development and reduced longevity when ablated during adult life, with the exception of *csw/SHP2* whose knockdown during adult life increased life span (Fig. 5A, 5C).

### Genome-wide RNAi Screen to Identify Modifiers of Phosphate-induced MAPK in *Drosophila* Hemocyte-like Cultured Cells

**Figure 6:**
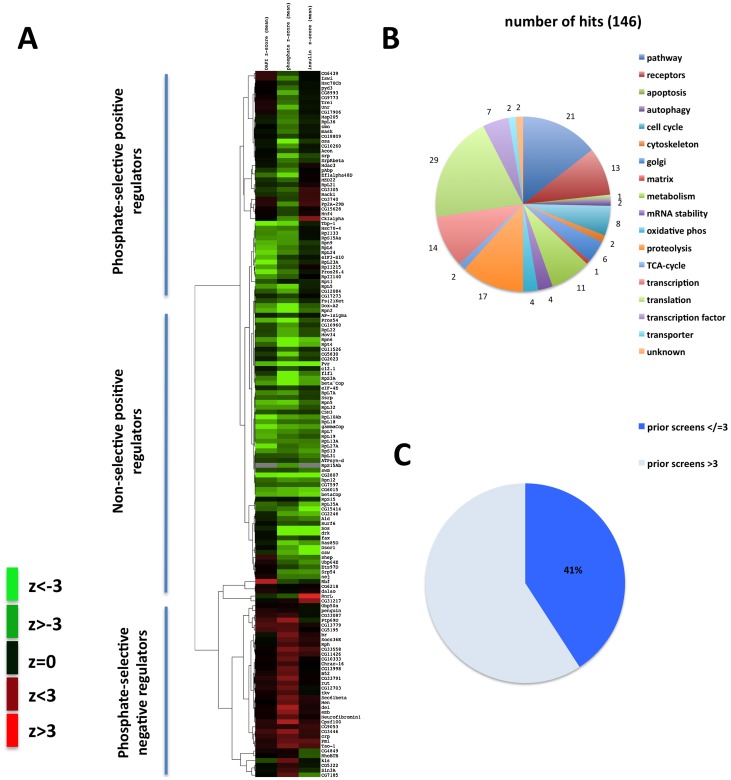
S2R+ genome-wide RNAi screen. A: heatmap of z-scores for 146 verified genes, cell count/well based on DAPI signal (first column), dpERK signal after 10 min. phosphate stimulation (middle column), and dpERK signal after 10 min. insulin stimulation (third column), green indicates positive regulators, red indicates negative regulators. **B:** Functional classification of 146 verified genes based on GO term categories using the DAVID tool (http://david.abcc.ncifcrf.gov/) [27], and FlyMine (www.flymine.org/) [28]. **C:** Number of genes identified in prior DRSC screens.

To identify novel components of the phosphate sensing pathway and to further understand whether the MAPK pathway mediates some of the phosphate effects observed on larval development, adult life span and hemolymph phosphate, we adapted a high throughput assay for MAPK activation in S2R+ cells [29] using 10 mM sodium phosphate as the stimulus and screened sixty-one 384-well plates with an average of 1.5 dsRNAs per gene in duplicate to cover the entire fly genome (approximately 14,000 genes). We obtained 1924 primary hits (see Methods). A subset of 555 genes was selected based on their expression in S2R+ cells, annotation and conservation in the human genome. These were re-screened *in vitro* using at least two independent dsRNAs targeting different regions of the mRNA encoding these genes. Further, as MAPK signaling is regulated by insulin signaling in *Drosophila* cells, we reasoned that selecting candidate genes that affect MAPK signaling under phosphate activation but not insulin would enrich for “phosphate-selective” regulators. Of a total of 146 genes verified with phosphate as the stimulus 84 did not score with insulin and are thus “phosphate-selective” in our assay conditions (Fig. 6A). From the remaining 62 non-selective genes we eliminated 43 “frequent hitters” in RNAi screens because they were identified in more than three prior DRSC screens and further evaluated 103 genes.

### Subsets of Phosphate-selective Genes are Modifiers of Larval Development, Life Span and Hemolymph Phosphate *in vivo*

**Figure 7:**
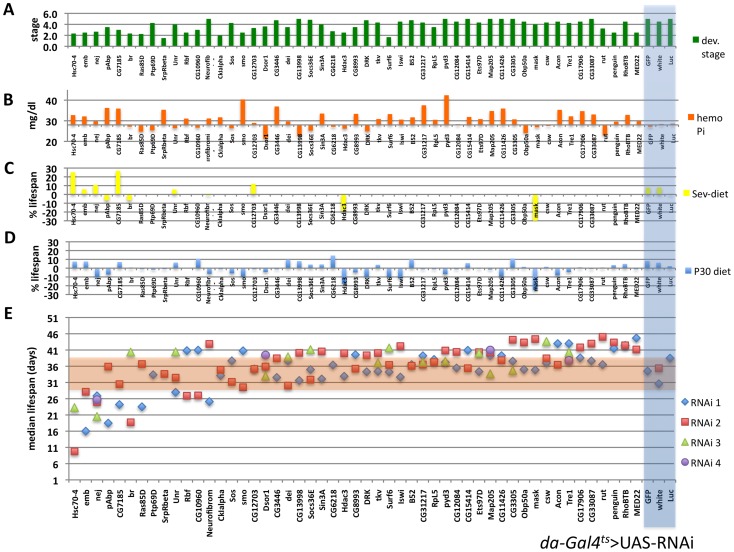
*In vivo* secondary screen using the *da*-*Gal4* ^ts^ as driver identifies genetic modifiers of *Drosophila* larval development, and adult hemolymph phosphate and lifespan. **A:** Developmental stage on standard medium, **B:** hemolymph phosphate concentration of young adult females cultured at 29°C for five days, and % change of life span on standard medium supplemented with 1% sevelamer (**C**), or 30 mM sodium phosphate (**D**) when compared to standard medium alone (**E**) (see also Table S1).

**Figure 8:**
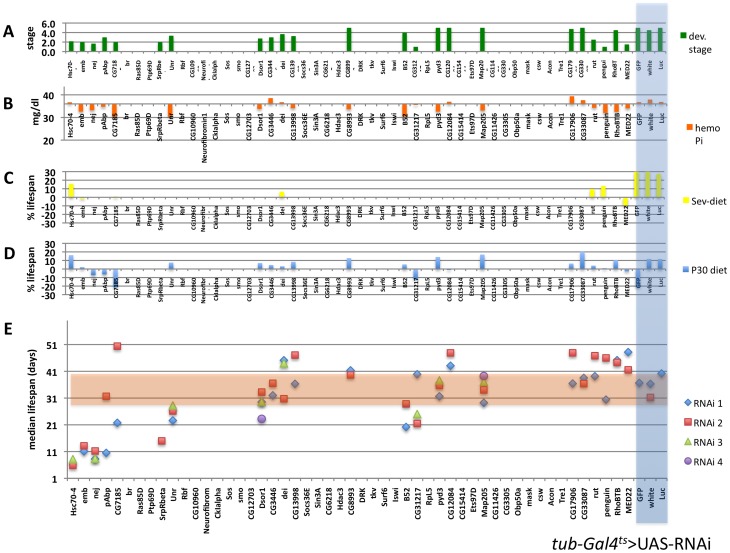
*In vivo* secondary screen using the *tub*-*Gal4* ^ts^ as driver identifies genetic modifiers of *Drosophila* larval development, and adult hemolymph phosphate and lifespan. A: Developmental stage on standard medium, **B:** hemolymph phosphate concentration of young adult females cultured at 29°C for five days, and % change of life span on standard medium supplemented with 1% sevelamer (**C**), or 30 mM sodium phosphate (**D**) when compared to standard medium alone (**E**) (see also Table S1).

To determine the effect of these103 genes *in vivo* we tested whether ubiquitous expression of RNAi constructs targeting these genes affected viability and if so, whether viability could be affected by dietary phosphate. Two or more RNAi lines were available from the TRiP and VDRC for 51 genes (including 40 ‘phosphate-selective’)**.** Viability tests were initially done using *da-Gal4* ^ts^ (Fig. 7) and a subset verified using *tub-Gal4* ^ts^ (Fig. 8).

When cultured at the inducing temperature of 29°C, knockdown of 22 genes resulted in lethality at or before puparation (Table 1, Fig. 7A). Lethality could not be rescued by supplementation of the media with 30 mM sodium phosphate (not shown). When reared at 18°C to keep the RNAi un-induced development was normal for most genes (Fig. S1A). When knockdown was induced within three days of eclosing ten genes prolonged median life span in adult males, when compared to control hairpins, while knockdown of seven genes reduced median life span (Table 1, Fig. 7E). RNAi knockdown of some genes furthermore influenced the life span responses to dietary phosphate, albeit non-significantly (Fig. 7C, 7D). Knockdown in young sibling females for five days identified seven negative regulators of hemolymph phosphate levels (i.e. knockdown resulted in increased levels), whereas knockdown of four genes resulted in decreased hemolymph phosphate (Table 1, Fig. 7B).

**Table 1:** Significant outliers in development, life span, and hemolymph phosphate.

Assay	Developmental lethal genes	Life span of adults (longer, shorter)	Hemolymph phosphate (higher, lower)
**Genes**	*CkIalpha, br, Hsc70-4, Hdac3, smo, Rbf,* *penguin, emb, CG6218, CG7185, rut,* *CG12703, RpL5, CG8993, dei, pAbp, Unr,* *mask, Sin3A, Ptp69D, CG3446, CG13998*	*RhoBTB, rut, CG33087, CG17906, * ***Tre1****, Acon,* ***pyd3, Map205*** *, RpL5, CG8993* *,* ***B52, CG3446*** *, * ***Unr*** *, CG7185, pAbp,* ***emb, Hsc70-4***	***pyd3, CG31217, CG3446*** *, pAbp, ****CG17906****,* *CG33087, Hsc70-4, nej,* *Socs36E, drk, * ***Dsor1***

*Drosophila* gene names for developmental lethal mutants and outliers in life span and hemolymph phosphate assays shown in Figures 6 and 7. Significance for longevity and hemolymph phosphate is based on Student’s t-test (p<0.05). Bold script indicates genes that remain significant after Bonferroni’s correction for multiple comparisons when we used p<0.003 for the life span assay based on 17 outliers and when we used p<0.007 and p<0.0125 for the hemolymph assay based on seven and four outliers, respectively. Underline script is used for longer/higher outliers, while regular script is used for shorter/lower outliers. (See Table S1 and Figs. 7, 8 for detailed results).

## Discussion

Using a cell-based MAPK assay, we identified 146 genes that are candidate positive and negative regulators of MAPK activation by phosphate *in vitro*. Among these are *drk/GRB2, sos, csw/SHP2, Ras85D, dos/Gab2,* and *dsor1/MEK*, confirming that the assay identified known members of the canonical MAPK-pathway. 61 (41%) of these genes had been identified in three or fewer of >40 genome-wide RNAi screens performed previously at the DRSC (Fig. 6C). Furthermore, non-selective genes verified in the current screen overlapped significantly with those identified in a previous RNAi screen for insulin-induced MAPK using S2R+ cells [29], supporting the idea that our approach selected for genes specific for the MAPK-pathway. Interestingly, knockdown of the 84 ‘phosphate-selective’ genes had little or no effect on cell number as indicated by a total fluorescence assay of cell number (Fig. 6A), whereas knockdown of most ‘non-selective’ regulators over 4 days reduced or increased cell number of the screening cell line. This finding may indicate that the ‘phosphate-selective’ set is enriched for genes distinct from the canonical MAPK pathway, which is known to regulate cell proliferation. Based on annotations, these genes have possible roles upstream of MAPK, as receptors, pathway components, or transcription factors (Fig. 6B). Finally, most are expressed ubiquitously, as expected for a metabolic phosphate sensing pathway (see cluster analysis based on expression data available in FlyAtlas [44] in Fig. S8).

We next demonstrated that larval development, hemolymph phosphate and adult life span are dependent on dietary phosphate. Dietary phosphate is required for larval development, while too much dietary phosphate reduces longevity of adult flies. Further, perturbation of the Malpighian tubules, the phylogenetic ancestor of renal tubules, in adult flies recapitulates some of the findings of CKD and elevates hemolymph phosphate and causes premature death. In addition, hyperphosphatemia resolves and life span is normal when these flies are cultured on medium supplemented with sevelamer that prevents absorption of phosphate from the diet. Finally, RNAi-mediated inhibition of MAPK-signaling affects larval development, and adult life span and hemolymph phosphate, raising the possibility that some *in vivo* effects involve activation of this signaling pathway by phosphate.

When characterizing 51 modifiers of activation of MAPK by phosphate *in vitro* we found that knockdown of 21 also affected larval development, adult hemolymph phosphate or life span (Table 1). Furthermore, knockdown of 15 genes had an effect consistent with the effect of knockdown of core pathway components *Ras85D, phl/D-Raf* or *Dsor1/MEK in vivo*. These genes are candidates that may link phosphate sensing to the MAPK pathway. Based on published literature the identified genes and their mammalian orthologs functionally group into 1) modifiers of MAPK-signaling *(embargoed (emb)* [45], [46], [47], *multiple ankyrin repeats single KH domain (MASK)* [48], [49]), 2) targets of phosphate (*Upstream of N-ras (Unr)* [50], [51], [52], [53], [54], [55], and *Trapped in endoderm 1 (Tre1)* [56]), 3) oxidative stress and aging (*Heat shock protein cognate 4 (Hsc70-4)* [57], [58], *CG8993* [59], [60], *Aconitase (Acon)* [61], [62], *CG3446* [63], B52 [64], [65]) and 4) skeletal growth and differentiation *(Smoothend (Smo)* [66], *CG33087 (LRP1)* [66], *Casein kinase 1 alpha (CkIalpha)* [66], *Histone deacetylase 3 (Hdac3)* [67], [68], [69], *Retinoblastoma-family protein (Rbf)* [70], [71], *Ribosomal protein L5 (RpL5)* [72], [73]). Future analysis will help determine whether they function upstream of MAPK or modify phosphate toxicity. Mammalian orthologs, if found to have similar roles, may facilitate the development of novel approaches to the management of hyperphosphatemia, for example in patients with CKD.

In summary, we established *Drosophila melanogaster* as a model system to study phosphate. By combining cell-based and *in vivo* RNAi-screening we identified a number of genes with putative and previously unrecognized roles in metabolic and homeostatic phosphate sensing.

## Supporting Information

Figure S1 **Developmental phenotypes. A:** Latest larval stage observed for F1 offspring generated in matings between 268 UAS-RNAi males and virgin *w-;tub-Gal80^ts20^;da-Gal4* females when cultured on standard medium at 18°C (non-inducing temperature). **B:** Latest larval stage observed for F1 offspring generated as described for (A) with the same genetic crosses on standard medium at 29°C (inducing temperature).
(TIF)

Figure S2 **The effect of sevelamer and PFA on larval development by developmental stage.** Shown is larval development of three wild-type strains: *y w* (YW), Oregon R (OR) *and* Canton S (CS) on control (C), P30, Sev1% and PFA 1 mM medium. Abbreviations as follows: ML, migrating instar 3 larva; PP, prepupa; P, pupa; M, adult male; F, adult female. Shown is one representative experiment with cumulative fly counts, means of three vials per condition.
(TIF)

Figure S3 **Effect of phosphate, sevelamer and PFA on adult life span. A:** Median life span of Canton S (CS) *and* Oregon R (OR) wild type males on standard medium or SM with 30 mM sodium phosphate or 1% sevelamer (CS: C, n = 155; P30, n = 62; Sev1, n = 58, OR: C, n = 117; P30, n = 52; Sev1, n = 59). **B:** Median life span of *y w* males on standard medium, supplemented with 15, 30 and 60 mM sodium phosphate (C, n = 550; P15, n = 282; P30, n = 465; P60, n = 115). To correct for the influence of osmolarity, life spans for P15, P30, and P60 are displayed as % of life spans for 15, 30, and 60 mM sodium sulfate, respectively.
(TIF)

Figure S4 **Dye uptake and excretion. A:** Uptake of food dyes within 60 min. **B:** Dye excretion over 60 min. after flies were loaded with food dyes over night. **C:** Ratio of amount of dyes present in excretions (theoretical ratio 5.2 from fresh food is indicated by blue line).
(TIF)

Figure S5 **Adult hemolymph Pi, Phosphate excretion and whole fly Pi.** Young adult *w^1118^*, Canton S or Oregon R females were cultured on standard medium alone or SM supplemented with 30 mM sodium phosphate (P30), or 1% sevelamer. Following culture for 5 days at 25°C, phosphate excretion was determined **(A**)(n = 3, 20 flies each), and hemolymph phosphate **(B)** (n = 3) and whole fly phosphate **(C)** (n = 10 individual flies) were measured.
(TIF)

Figure S6 **Genome-wide RNAi screen (Controls). 20,000 S2R+ cells/384 well were treated with 0.375 ug dsRNA targeting Lac-Z, Ras85D, Dsor1/MEK, Rho1 and thread (A,** primary screen) or LacZ, drk/GRB2, and Tao-1 **(B,** secondary screen)/well as described in materials and methods. After four days 10 mM phosphate (P10) **(A, B),** 50 ug/ml human insulin **(C)** or 30 uM of the MEK-inhibitor UO126 (UO126) were added for 10 min., followed by fixation and antibody staining for dpERK and cellular staining with Dead Red or DAPI and detection of total fluorescence with the appropriate filter sets. Ratios of dpERK signal over cell Dead Red or nuclear DAPI stain were expressed after subtraction of well background and wandering-median correction across each 384-well plate as z-scores.
(TIF)

Figure S7 **P-induced ERK activation in murine and** ***Drosophila*** **cells is blocked by RNAi-knockdown of sodium-phosphate co-transporters and members of the canonical MAPK pathway.** RNAi knockdown in S2R+ cells using dsRNA targeting luciferase (luc), insulin receptor (IR), two sodium-phosphate co-transporters (MFS10 and MFS13), or various components of the canonical MAPK pathway was performed for three days prior to challenge with 10 mM sodium phosphate (pH7.4) or 25 ug/ml Insulin for 3 min. Immunoblot analysis of cell lysates was performed with anti-dpERK antibody, converted into percent-stimulation (mean+/− SD of three independent experiments).
(TIF)

Figure S8 **Tissue distribution of genes indentified in the primary screen.** Available expression data for all 146 genes were downloaded from Fly Atlas [44], normalized by gene and hierarchically clustered using Cluster 3.0 [32] and displayed using Java TreeView 1.1.6 [33]. Red indicates high, green low expression.
(TIF)

Figure S9 **Temperature dependence of RNAi-effects on hemolymph Phosphate and adult life span. A:** Hemolymph phosphate after culture of F1 offspring at inducing temperature 29°C (orange bars), and 18°C (blue line) for 63 RNAi-lines, control hairpins are shown in light orange, mean+/−SEM. **B, C:** Median life-spans of 118 and 68 RNAi-lines at inducing temperature 29°C (orange bars) and 18°C (blue line)
(TIF)

Table S1 **Annotations of 146 validated genes.**
(XLSX)

Methods S1 **Supplemental methods.**
(DOCX)
