# Supplementary figures and images for: Genetic Determinants of Phosphate Response in Drosophila
Source: PLoS One. 2013 Mar 8;8(3):e56753. doi: 10.1371/journal.pone.0056753 (PMC3592877; doi:10.1371/journal.pone.0056753)

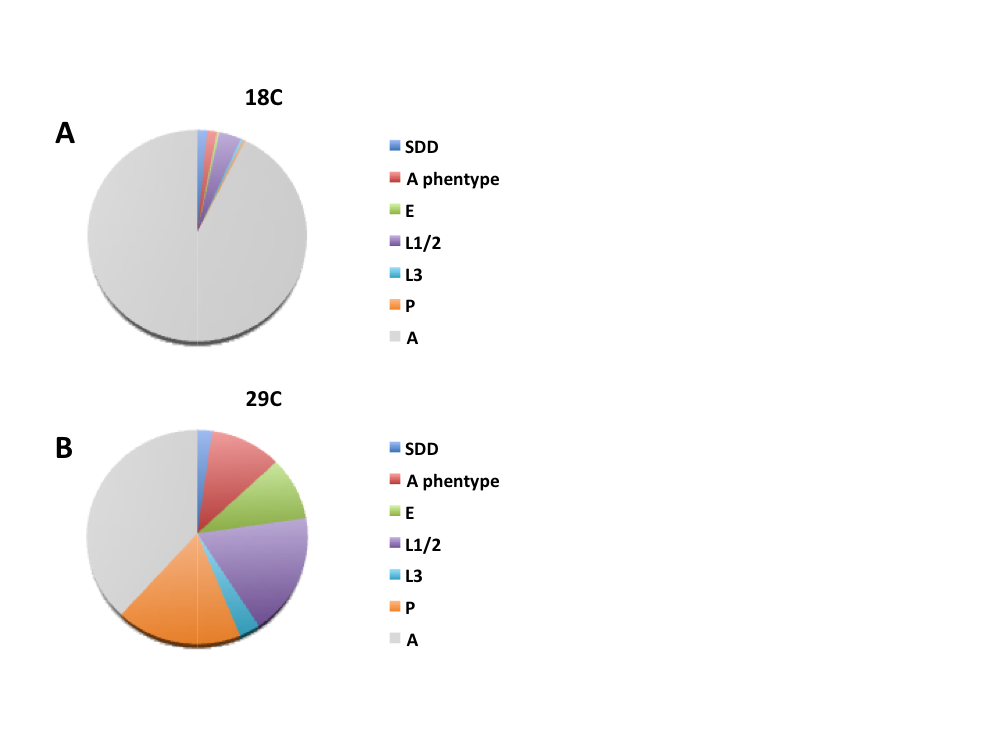

Supplement: Figure S1 — Developmental phenotypes. A: Latest larval stage observed for F1 offspring generated in matings between 268 UAS-RNAi males and virgin w-;tub-Gal80ts20;da-Gal4 females when cultured on standard medium at 18°C (non-inducing temperature). B: Latest larval stage observed for F1 offspring generated as described for (A) with the same genetic crosses on standard medium at 29°C (inducing temperature). (TIF) [file pone.0056753.s001.tif]

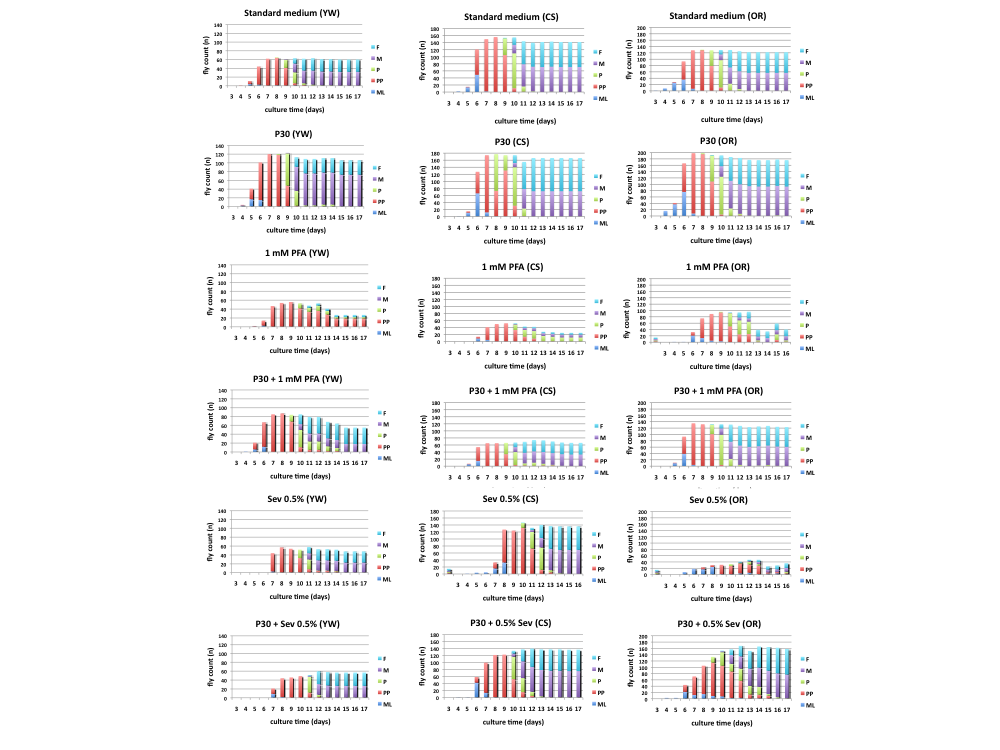

Supplement: Figure S2 — The effect of sevelamer and PFA on larval development by developmental stage. Shown is larval development of three wild-type strains: y w (YW), Oregon R (OR) and Canton S (CS) on control (C), P30, Sev1% and PFA 1 mM medium. Abbreviations as follows: ML, migrating instar 3 larva; PP, prepupa; P, pupa; M, adult male; F, adult female. Shown is one representative experiment with cumulative fly counts, means of three vials per condition. (TIF) [file pone.0056753.s002.tif]

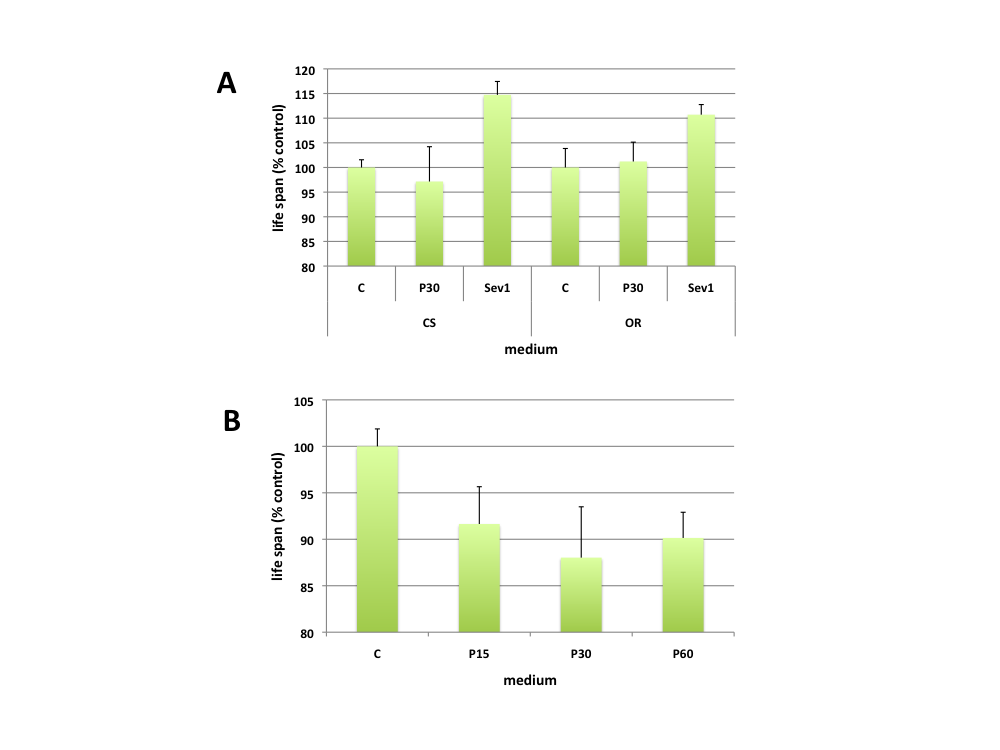

Supplement: Figure S3 — Effect of phosphate, sevelamer and PFA on adult life span. A: Median life span of Canton S (CS) and Oregon R (OR) wild type males on standard medium or SM with 30 mM sodium phosphate or 1% sevelamer (CS: C, n = 155; P30, n = 62; Sev1, n = 58, OR: C, n = 117; P30, n = 52; Sev1, n = 59). B: Median life span of y w males on standard medium, supplemented with 15, 30 and 60 mM sodium phosphate (C, n = 550; P15, n = 282; P30, n = 465; P60, n = 115). To correct for the influence of osmolarity, life spans for P15, P30, and P60 are displayed as % of life spans for 15, 30, and 60 mM sodium sulfate, respectively. (TIF) [file pone.0056753.s003.tif]

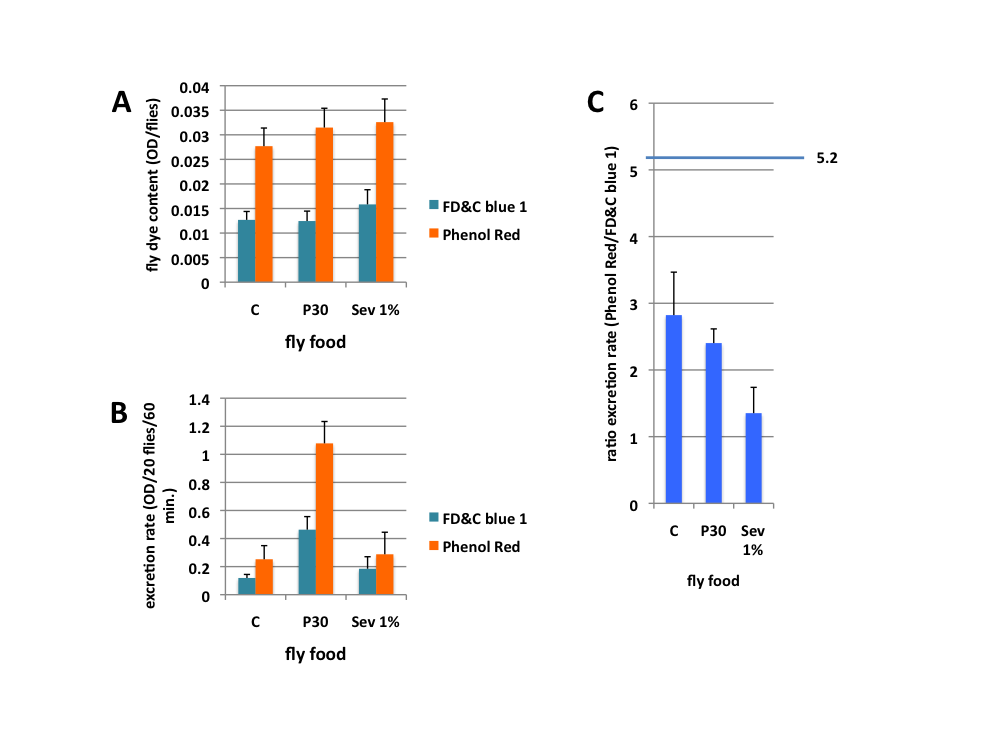

Supplement: Figure S4 — Dye uptake and excretion. A: Uptake of food dyes within 60 min. B: Dye excretion over 60 min. after flies were loaded with food dyes over night. C: Ratio of amount of dyes present in excretions (theoretical ratio 5.2 from fresh food is indicated by blue line). (TIF) [file pone.0056753.s004.tif]

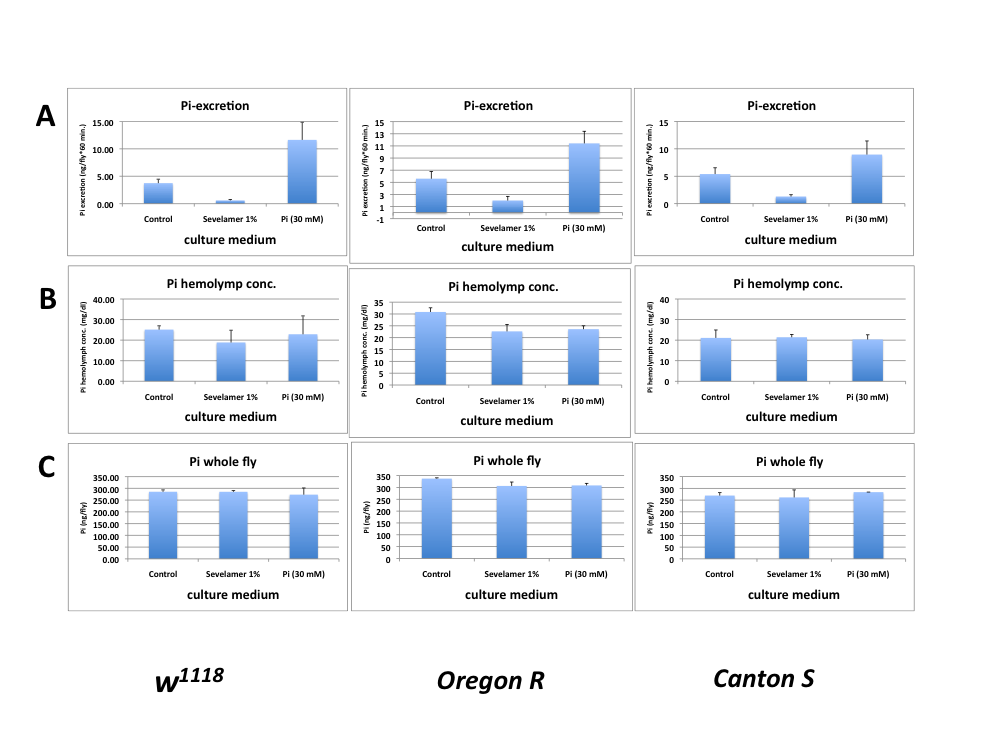

Supplement: Figure S5 — Adult hemolymph Pi, Phosphate excretion and whole fly Pi. Young adult w1118, Canton S or Oregon R females were cultured on standard medium alone or SM supplemented with 30 mM sodium phosphate (P30), or 1% sevelamer. Following culture for 5 days at 25°C, phosphate excretion was determined (A)(n = 3, 20 flies each), and hemolymph phosphate (B) (n = 3) and whole fly phosphate (C) (n = 10 individual flies) were measured. (TIF) [file pone.0056753.s005.tif]

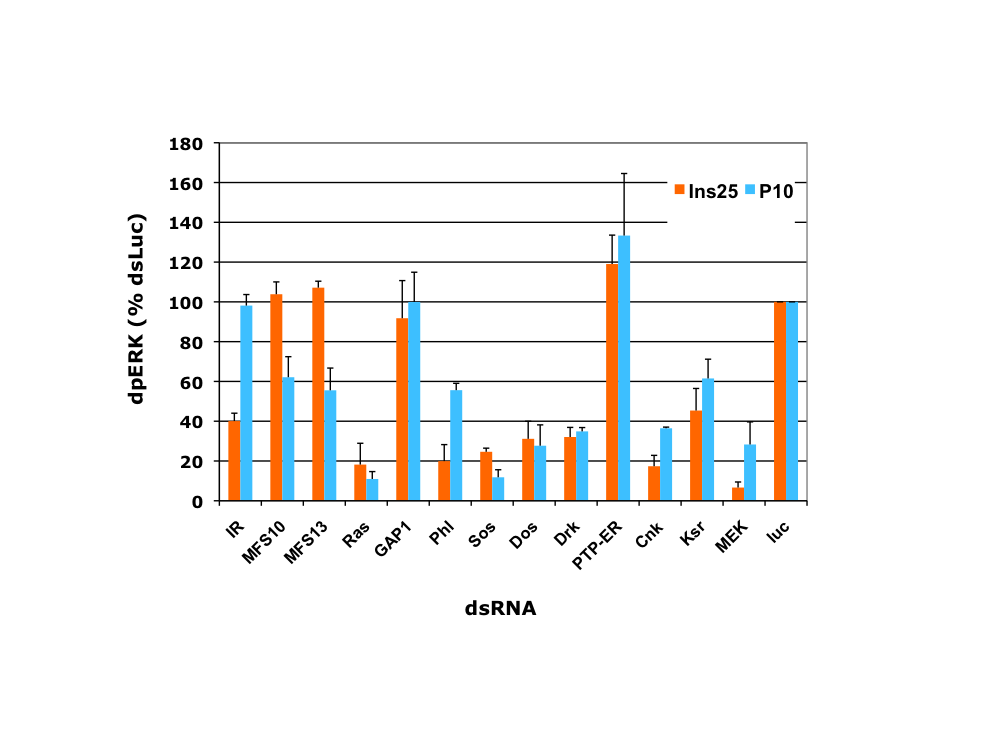

Supplement: Figure S7 — P-induced ERK activation in murine and Drosophila cells is blocked by RNAi-knockdown of sodium-phosphate co-transporters and members of the canonical MAPK pathway. RNAi knockdown in S2R+ cells using dsRNA targeting luciferase (luc), insulin receptor (IR), two sodium-phosphate co-transporters (MFS10 and MFS13), or various components of the canonical MAPK pathway was performed for three days prior to challenge with 10 mM sodium phosphate (pH7.4) or 25 ug/ml Insulin for 3 min. Immunoblot analysis of cell lysates was performed with anti-dpERK antibody, converted into percent-stimulation (mean+/− SD of three independent experiments). (TIF) [file pone.0056753.s007.tif]

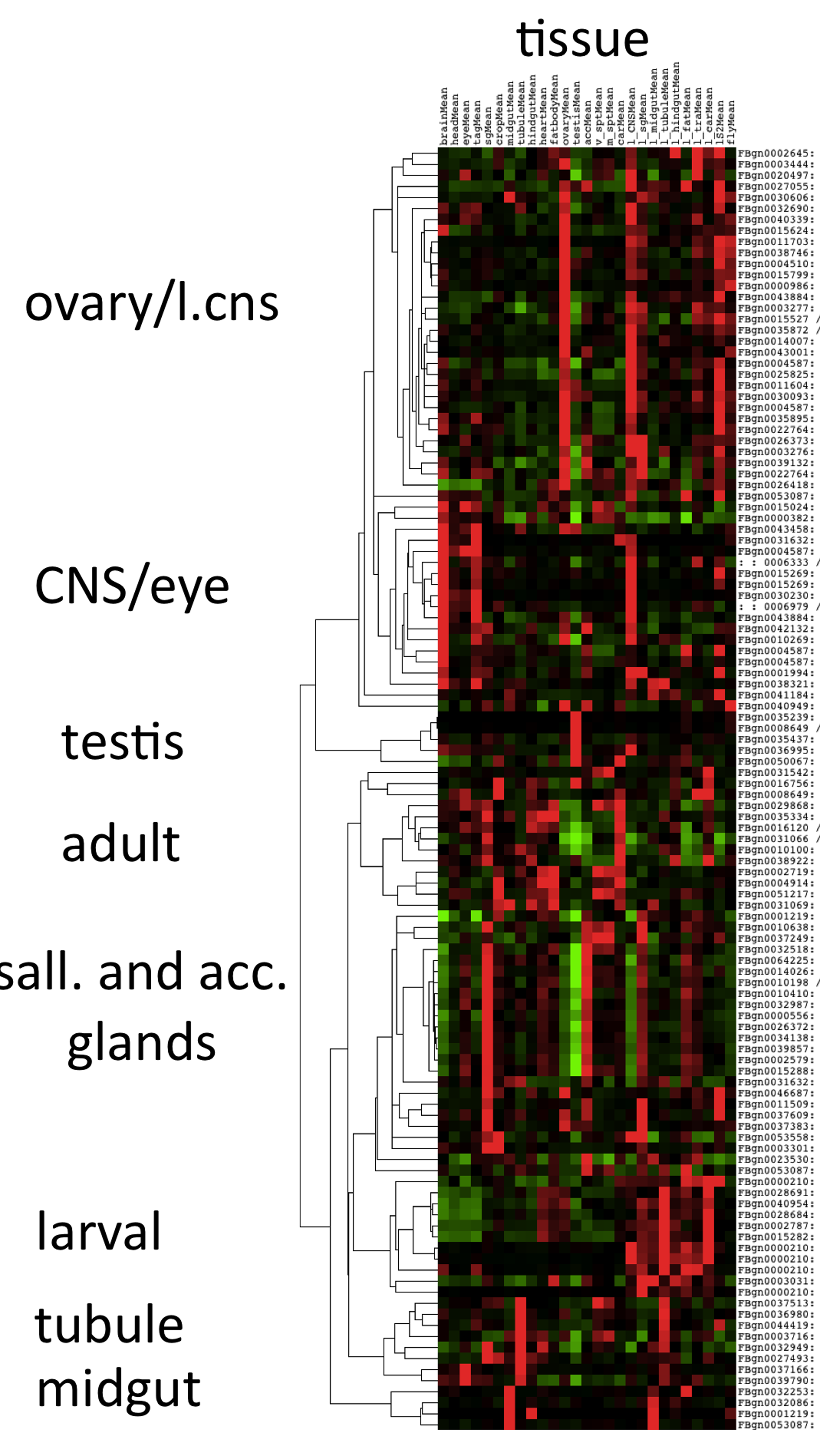

Supplement: Figure S8 — Tissue distribution of genes indentified in the primary screen. Available expression data for all 146 genes were downloaded from Fly Atlas [44], normalized by gene and hierarchically clustered using Cluster 3.0 [32] and displayed using Java TreeView 1.1.6 [33]. Red indicates high, green low expression. (TIF) [file pone.0056753.s008.tif]

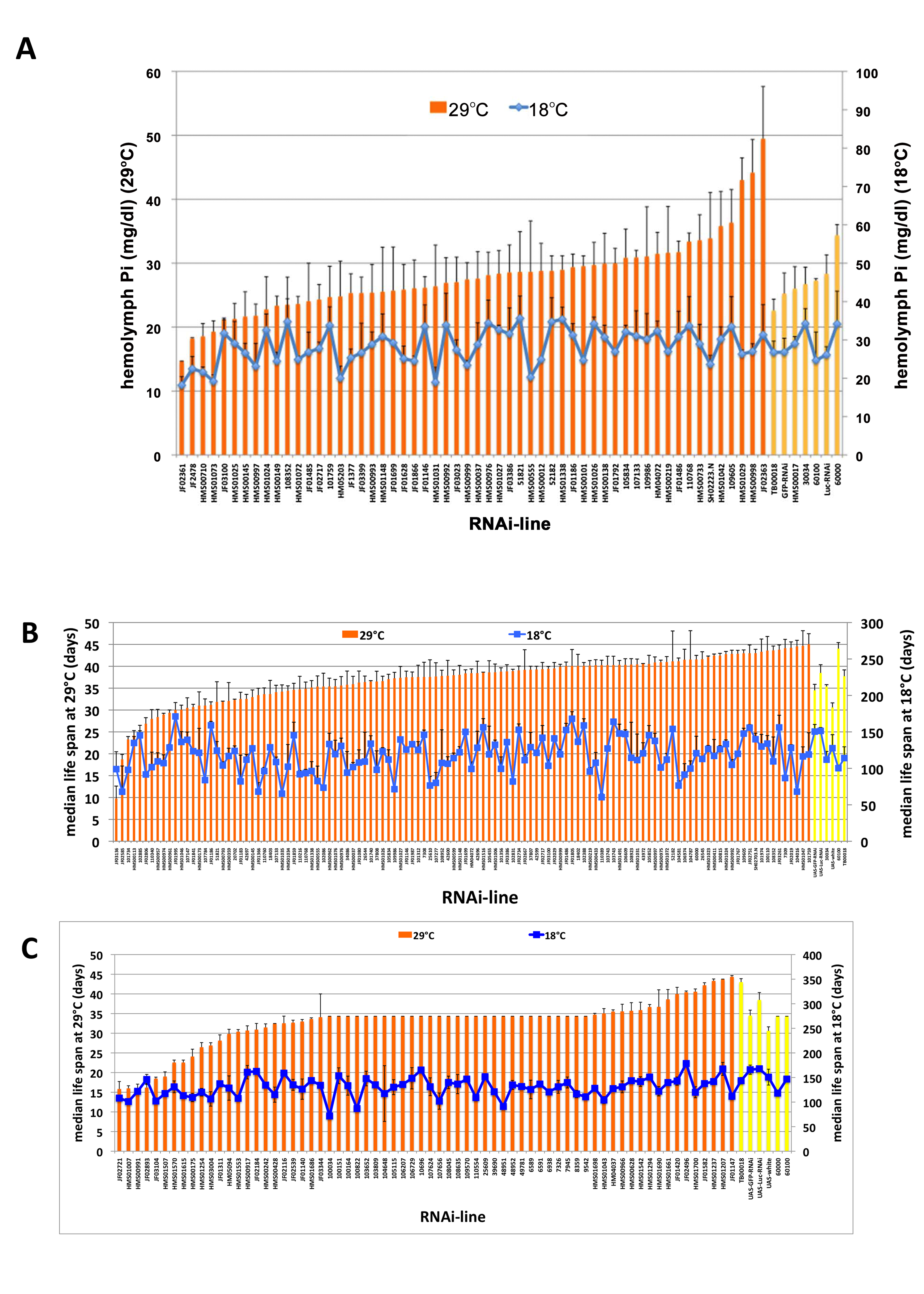

Supplement: Figure S9 — Temperature dependence of RNAi-effects on hemolymph Phosphate and adult life span. A: Hemolymph phosphate after culture of F1 offspring at inducing temperature 29°C (orange bars), and 18°C (blue line) for 63 RNAi-lines, control hairpins are shown in light orange, mean+/−SEM. B, C: Median life-spans of 118 and 68 RNAi-lines at inducing temperature 29°C (orange bars) and 18°C (blue line) (TIF) [file pone.0056753.s009.tif]
