# Supplementary material for: Genetic Determinants of Phosphate Response in Drosophila
Source: PLoS One. 2013 Mar 8;8(3):e56753. doi: 10.1371/journal.pone.0056753 (PMC3592877; doi:10.1371/journal.pone.0056753)
Supplement: Methods S1 — Supplemental methods. (DOCX) [file pone.0056753.s011.docx]

**Supplemental Methods**

**Fly stocks and culture**

Additional stocks used in the study include *yellow white* (*yw*), Canton S (BSDC stock ID #1), *w^1118^*, and Oregon R, and *w-;Uro-Gal4/flCyO;tub-Gal80^ts7^ (Uro-Gal4^ts^, w-;Uro-Gal4/CyO* to generate this stock was kindly provided by Julian Dow, University of Glasgow, UK). Transgenic flies carrying RNAi constructs were obtained from the Transgenic RNAi Project (TRiP) [74] and Vienna Drosophila RNAi Center (VDRC) [75]. Additional stocks used: *w-, UAS-rpr(I)*(Bloomington),

Standard fly culture was performed at 25°C on 17 g/l yeast, 9.8 g/l soy flour, 71 g/l corn meal, 5.6 g/l agar, 5.6 g/l malt, 75 ml/l corn syrup, 4 ml/l propionic acid and 250 mg/l Tegosept (Spectrum M1187). This standard medium (SM) was supplemented with 30 mM sodium phosphate (pH 6.0)(P30), 30 mM sodium sulfate (pH 6.0)(S30), 0.1-10 mM phosphonoformic acid (Sigma P6801)(PFA0.1-10), or 0.1-1% sevelamer (gift from Dr. Yves Sabbagh, Genzyme, Inc.)(Sev0.1-1%).

Supplementation of SM with 30 mM sodium sulfate was tolerated, while 30 mM sodium phosphate reduced life span. However, 60 mM sodium sulfate did reduce life span (Supplemental Fig. S3B), which is why 30 mM sodium phosphate was used for all subsequent experiments.

Defined medium (DM) was prepared according to Roberts [22] in which casein as a protein source is replaced with egg whites (30 g/l agar, 55 g/l egg white (MP Biomedicals 901633), 7.5 g/l sucrose (Sigma S0389), 0.3 g/l cholesterol (Sigma C3045), 0.24 g/l choline chloride (Acros 110290500), 0.64 g/l inosine (MP 102049), 0.57 g/l uridine (Alfa Aesar A15227), 1 g/l NaHCO_2_ (Sigma S5761), 0.71 g/l KH_2_PO_4_ (Fisher BP362-500), 3.73 g/l K_2_HPO_4_ (Fisher BP363-500), 0.62 g/l MgSO_4_x7H_2_O (Sigma M9397), 0.1218 g/l K_2_SO_4_ (Sigma P0772), and a multivitamin mixture containing 0.002 g/l thiamine (MP Biomedicals 194749), 0.01 g/l riboflavine (Sigma R9504), 0.012 g/l nicotinic acid (Acros 128290050), 0.016 g/l Ca-pantothenate (Acros 243300050), 0.0025 g/l pyridoxine (MP Biomedicals 194733), 0.0002 g/l biotin (Acros 230095000), 0.003 g/l folic acid (MP Biomedicals 101725)). We added 0.003% propionic acid (Malincrodt 7179-04) and 1 mg/ml Tegosept (Spectrum M1187) to inhibit fungal growth.

To quantify larval development on different media, we prepared culture vials with SM and vehicle or supplements as described above. Care was taken to avoid variation in fluid content. Specifically, all supplements were premixed as 10X aqueous solutions and added to SM to a final concentration of 1X in 5 ml SM/culture vial).

**Dye-feeding assay**

The dye-feeding assay was modified from Edgecomb et al. (1994). Briefly, male

flies aged 3–10 days were switched from SM to SM supplemented with 1% FD&C Blue No. 1 (Sigma) and 1% Phenol Red (Sigma) for 1 hr or 18 h at 25°C. As described by Edgecomb et al. [24], FD&C Blue No. 1 remains in the digestive tract and is not affected by gut pH and enzymes, whereas Phenol Red is absorbed and excreted with the urine. To determine the dye consumption rate after feeding for 60 min., flies were transferred to SM w/o dyes for 60 min. Fly heads were removed to prevent eye pigment from interfering with measurement of the dye. Fly bodies were individually homogenized using a Kontes pestle homogenizer in 100 ul water. To evaluate dye excretion rate, flies fed on SM with dye overnight were transferred to SM for 60 min., followed by 60 min. collection of blue/red excretions in a 1.5 ml vial. Flies were discarded and excretions dissolved in 100 ul water prior to measurement. Cleared supernatants were measured at 430 nm (phenol red) and 630 nm (FD&C blue-1) using a Nano-drop 6000 (Fisher). A theoretical ratio of 5.2 (OD430/630 nm) is expected, if no additional Phenol Red was excreted with urine, whereas ratios of more than 5.2 may indicate excretion of Phenol Red in excess of the amount present in the feces.

**Genome-wide *Drosophila* cell-based RNAi screen**

The genome-wide dsRNA screening collection of the *Drosophila* RNAi Screening Center (DRSC) covering >95% of the *Drosophila* genome has been described previously [25]. This library contains one or two unique dsRNAs per gene for approximately 14,000 genes. The library was designed based on current rules for minimizing off-target effects and is annotated based on a recent FlyBase release (see also [www.flyrnai.org](http://www.flyrnai.org)). Our assay was based on the assay used in a previous screen using insulin, EGF and PVF as stimulus [29]. Briefly, two copies each of sixty-two unique 384-well plates pre-aliquoted with the DRSC library were seeded with S2R+ cells, a variant adherent S2 line [26], at 20,000 cells/well and incubated in serum-free medium for 45 minutes. Next, serum-containing medium was added and the cells were incubated for four days at 25ºC. On day four, 60 mM Na-phosphate buffer (pH 7.4) was added in 10 ul/well for a final concentration of 10 mM. Following stimulation at 25ºC for 10 min., cells were fixed by the addition of 10 ul/well of 28% formaldehyde for a final concentration of 4% for 10 minutes and washed twice with PBS+0.1%Triton (PBST). A monoclonal antibody directed against dual-phosphorylated mammalian ERK1/2 was obtained from Cell Signaling Technologies, Inc (#CS4370; see also [29]). The antibody was added in PBST+3% bovine serum albumin (BSA) at a final concentration of 1:400 in 10 ul/well. After incubation for 18-24 hrs at 4ºC, the wells were rinsed three times with 50 ul PBST, followed by incubation with PBST+3% BSA containing an anti-rabbit secondary antibody directly conjugated to Alexa 647 (Invitrogen #A21246, working concentration 1:2000) and 1 ng/ml DAPI (Roche #10 236 276 001) at room temperature for 2 hrs. In initial experiments, Dead Red (Molecular Probes Kit# L7013, Invitrogen) was used as a protein stain according to the manufacturer’s instructions to validate cell counts obtained with DAPI. Following three additional rinses with PBST, the plates were read in 30 ul PBST/well using a Molecular Devices Analyst GT micro-well plate fluorometer and Typhoon 9410 Imager (Amersham/ GE Healthcare) to detect phospho-ERK, protein and DNA content. Knockdown of *Ras85D, and Dsor1/MEK* upstream of ERK reduced fluorescence, whereas knockdown of *Rho* increased fluorescence in control wells of each assay plate, supporting the idea that the screen assay was working as expected. The chemical MEK inhibitor UO126 (final concentration 30 uM), and *thread* RNAi which causes cell death were used as an additional controls on each assay plate (Fig. S-6A-C).

**Primary screen data analysis**

Our data analysis pipeline was directed towards removing as many sources of noise as possible. Many of the issues we faced have been recently reviewed [76] and variations of the approaches described in the review were applied to this work. Raw dpERK, DAPI and Dead Red intensities were first background subtracted to account for autofluorescence of the plastic screening plates. To correct for spatial irregularities in the fluorescence measurements across individual screening plates (*e.g.* “edge-effects”), dpERK values were corrected by dividing by the moving median of each well’s column and row. Corrected dpERK values were then divided by corrected DAPI or Dead Red values for each channel to yield a normalized dpERK value. Median and IQR of each 384-well plate were used for Z-score transformation of normalized corrected dpERK values, an approach that is less sensitive to outliers than use of standard deviation and means. Z-scores for replicate wells for each unique dsRNA were then averaged. Genes whose average Z-scores met a cutoff of +/-1.0 with at least one of the targeting RNAi-amplicons were subjected to further evaluation using option #1 of the screen analysis tool RNAi-Cut (<http://groups.csail.mit.edu/cb/RNAiCut/>) [77], which automatically defines a cut-off depending on hit-connectivity using protein-protein interaction (PPI) networks and lowered the final z-score cut-off to approximately +/- 1.3. By comparison, control dsRNAs targeting *drk/GRB2, and Tao1*, as well as the chemical MEK inhibitor UO126 (30 uM) achieved larger z-scores (Supplemental Fig. S6B-C). We next annotated our hits list for expression in the screening cell line, molecular function, conservation, and other features by cross-referencing with publicly-available databases i.e. DAVID [27], fly-MINE [28] and manual curation of the list as described in Methods. Care was taken to use follow-up dsRNAs without significant predicted off-target effects (OTEs) [78,79]. To identify targets that might preferentially regulate phosphate-induced MAPK, in the validation round, we assayed 12 replicate plates each with cells treated with 10 mM Na-phosphate buffer or with 25 ug/ml human insulin as stimulus. After wandering median correction, corrected normalized dpERK values were again transformed into z-scores as above and subjected to a two-sided t-test. Of a total of 555 genes 146 could be verified at p<0.05. Of these, 84 preferentially interfered with phosphate-induced MAPK at the concentrations used. From the remaining 62 non-selective genes we eliminated 43 “frequent hitters” in RNAi screens because they were identified in more than three of 44 prior DRSC screens and further evaluated 103 genes.

***in vivo* *Drosophila* RNAi screen**

Transgenic RNAi lines targeting 103 genes were obtained from the Transgenic RNAi Project (TRiP, http://www.flyrnai.org/TRiP-HOME.html) and the Vienna Drosophila RNAi Center (VDRC, <http://stockcenter.vdrc.at/control/main>). The “virginator” stock *w-, hs-hid(y)/w-;tub-Gal80^ts20^;da-Gal4* was used to obtain large numbers of virgin females for the initial screen that were crossed with transgenic males from the TRiP or VDRC collections. F1 offspring of the genotypes: *UAS-RNAi/w-;tub-Gal80^ts20^/+;da-Gal4/+, w-;UAS-RNAi/tub-Gal80^ts20^;daGal4/+, or w-;tub-Gal80^ts20^/+;UAS-RNAi/da-Gal4* were then used for subsequent experiments and hereafter, will be referred to using simply the “driver>RNAi line ID or target gene name” convention. The TRiP collection used three different RNAi vectors for hairpin expression in the soma: valium 10 contains long dsRNAs with homology to the targeted gene over several hundred base pairs, while valium 20 and 22 contain short hairpins containing a 21 bp homology domain for the target gene. All vectors were inserted in a single AttP2 site on chromosome 3. The VDRC library uses long dsRNAs in a modified pUAST vector, pMF3 (“GD” library; randomly integrated), or pKC26 and 43 (“KK” library; inserted in a single AttP VIE-260B landing site on chromosome 2). Hairpin effects were comparable between males and females, and all experiments were repeated at 18˚C to show that effects observed at the inducing temperature were specific to the RNAi (Fig. S1A, Fig. S9).

Results with additional control lines were: Life span of F1 males generated with the attP2 landing site strain *y sc v* (TRiP #TB00018) was 38+/-1.4 days. Life span of F1 males generated with *w^1118^* isogenic flies (VDRC #60000) was 42+/-0.9 days, and of *y w[1118];P{attP,y[+],w[3`]* (VDRC #60100) was 43+/-1.4 days. Median±IQR of median life spans across the entire screen for F1 males generated with TRiP lines was 34+/-8 days (n=165, 15794 flies), for VDRC GD lines was 38+/-5 days (n=34, 2450 flies), and for VDRC KK lines was 40+/-3 days (n=53, 3554 flies).

Hemolymph phosphate of F1 generation females for the control strain *y sc v* (TRiP TB00018) was 23.5+/-1.5 mg/dl. Hemolymph phosphate of F1 females of *w^1118^* isogenic females (VDRC #60000) was 32+/-1.2 mg/dl, and of *y w[1118];P{attP,y[+],w[3`]* (VDRC #60100) was 30+/-1.3 mg/dl. Median±IQR of hemolymph concentrations across the entire screen for F1 females generated with TRIP lines was 29+/-6 mg/dl (n=146), for VDRC-GD lines was 32+/-5 mg/dl (n=32), and for VDRC-KK lines was 32+/-5 mg/dl (n=53).

**References**

1. Bevington A, Kemp GJ, Graham R, Russell G (1992) Phosphate-sensitive enzymes: possible molecular basis for cellular disorders of phosphate metabolism. Clin Chem Enzym Comms 4: 235-257.

2. Oshima Y (1997) The phosphatase system in Saccharomyces cerevisiae. Genes Genet Syst 72: 323-334.

3. Lenburg ME, O'Shea EK (1996) Signaling phosphate starvation. Trends Biochem Sci 21: 383-387.

4. Kaffman A, Herskowitz I, Tjian R, O'Shea EK (1994) Phosphorylation of the transcription factor PHO4 by a cyclin-CDK complex, PHO80-PHO85. Science 263: 1153-1156.

5. Khoshniat S, Bourgine A, Julien M, Weiss P, Guicheux J, et al. (2010) The emergence of phosphate as a specific signaling molecule in bone and other cell types in mammals. Cell Mol Life Sci 68: 205-218.

6. Bergwitz C, Juppner H (2011) Phosphate sensing. Adv Chronic Kidney Dis 18: 132-144.

7. Yamazaki M, Ozono K, Okada T, Tachikawa K, Kondou H, et al. (2010) Both FGF23 and extracellular phosphate activate Raf/MEK/ERK pathway via FGF receptors in HEK293 cells. J Cell Biochem 111: 1210-1221.

8. Mansfield K, Teixeira CC, Adams CS, Shapiro IM (2001) Phosphate ions mediate chondrocyte apoptosis through a plasma membrane transporter mechanism. Bone 28: 1-8.

9. Yoshiko Y, Candeliere GA, Maeda N, Aubin JE (2007) Osteoblast autonomous Pi regulation via Pit1 plays a role in bone mineralization. Mol Cell Biol 27: 4465-4474.

10. Bergwitz C, Rasmussen MD, Derobertis C, Wee MJ, Sinha S, et al. (2012) Roles of major facilitator superfamily transporters in phosphate response in Drosophila. PLoS One 7: e31730.

11. Beck GR, Jr., Knecht N (2003) Osteopontin regulation by inorganic phosphate is ERK1/2-, protein kinase C-, and proteasome-dependent. J Biol Chem 278: 41921-41929.

12. Nair D, Misra RP, Sallis JD, Cheung HS (1997) Phosphocitrate inhibits a basic calcium phosphate and calcium pyrophosphate dihydrate crystal-induced mitogen-activated protein kinase cascade signal transduction pathway. J Biol Chem 272: 18920-18925.

13. Julien M, Magne D, Masson M, Rolli-Derkinderen M, Chassande O, et al. (2007) Phosphate stimulates matrix Gla protein expression in chondrocytes through the extracellular signal regulated kinase signaling pathway. Endocrinology 148: 530-537.

14. Chang SH, Yu KN, Lee YS, An GH, Beck GR, Jr., et al. (2006) Elevated inorganic phosphate stimulates Akt-ERK1/2-Mnk1 signaling in human lung cells. Am J Respir Cell Mol Biol 35: 528-539.

15. Bringhurst FR, Leder BZ (2006) Regulation of calcium and phosphate homeostasis. In: DeGroot LJ, Jameson JL, editors. Endocrinology. Fifth ed. Philadelphia: W.B. Saunders Co. pp. 805-843.

16. Zhang MY, Ranch D, Pereira RC, Armbrecht HJ, Portale AA, et al. (2012) Chronic Inhibition of ERK1/2 Signaling Improves Disordered Bone and Mineral Metabolism in Hypophosphatemic (Hyp) Mice. Endocrinology 153: 1806-1816.

17. Bergwitz C, Juppner H (2009) Disorders of Phosphate Homeostasis and Tissue Mineralisation. Endocr Dev 16: 133-156.

18. Sprecher E (2010) Familial tumoral calcinosis: from characterization of a rare phenotype to the pathogenesis of ectopic calcification. J Invest Dermatol 130: 652-660.

19. Mizobuchi M, Towler D, Slatopolsky E (2009) Vascular calcification: the killer of patients with chronic kidney disease. J Am Soc Nephrol 20: 1453-1464.

20. Gutierrez OM, Mannstadt M, Isakova T, Rauh-Hain JA, Tamez H, et al. (2008) Fibroblast growth factor 23 and mortality among patients undergoing hemodialysis. N Engl J Med 359: 584-592.

21. Morishita K, Shirai A, Kubota M, Katakura Y, Nabeshima Y, et al. (2001) The progression of aging in klotho mutant mice can be modified by dietary phosphorus and zinc. J Nutr 131: 3182-3188.

22. Roberts DM (1998) Drosophila: A Practical Approach (The Practical Approach Series). New York: Oxford University Press.

23. Chertow GM, Dillon M, Burke SK, Steg M, Bleyer AJ, et al. (1999) A randomized trial of sevelamer hydrochloride (RenaGel) with and without supplemental calcium. Strategies for the control of hyperphosphatemia and hyperparathyroidism in hemodialysis patients. Clin Nephrol 51: 18-26.

24. Edgecomb RS, Harth CE, Schneiderman AM (1994) Regulation of feeding behavior in adult Drosophila melanogaster varies with feeding regime and nutritional state. J Exp Biol 197: 215-235.

25. Flockhart IT, Booker M, Hu Y, McElvany B, Gilly Q, et al. (2012) FlyRNAi.org--the database of the Drosophila RNAi screening center: 2012 update. Nucleic Acids Res 40: D715-719.

26. Yanagawa S, Lee JS, Ishimoto A (1998) Identification and characterization of a novel line of Drosophila Schneider S2 cells that respond to wingless signaling. J Biol Chem 273: 32353-32359.

27. Huang da W, Sherman BT, Lempicki RA (2009) Systematic and integrative analysis of large gene lists using DAVID bioinformatics resources. Nat Protoc 4: 44-57.

28. Lyne R, Smith R, Rutherford K, Wakeling M, Varley A, et al. (2007) FlyMine: an integrated database for Drosophila and Anopheles genomics. Genome Biol 8: R129.

29. Friedman A, Perrimon N (2006) A functional RNAi screen for regulators of receptor tyrosine kinase and ERK signalling. Nature 444: 230-234.

30. McGuire SE, Mao Z, Davis RL (2004) Spatiotemporal gene expression targeting with the TARGET and gene-switch systems in Drosophila. Sci STKE 2004: pl6.

31. Dunn OJ (1961) Multiple Comparisons Among Means. Journal of the American Statistical Association 56: 52-64.

32. Eisen MB, Spellman PT, Brown PO, Botstein D (1998) Cluster analysis and display of genome-wide expression patterns. Proc Natl Acad Sci U S A 95: 14863-14868.

33. Page RD (1996) TreeView: an application to display phylogenetic trees on personal computers. Comput Appl Biosci 12: 357-358.

34. Hu Y, Flockhart I, Vinayagam A, Bergwitz C, Berger B, et al. (2011) An integrative approach to ortholog prediction for disease-focused and other functional studies. BMC Bioinformatics 12: 357.

35. Eppig JT, Blake JA, Bult CJ, Kadin JA, Richardson JE (2012) The Mouse Genome Database (MGD): comprehensive resource for genetics and genomics of the laboratory mouse. Nucleic Acids Res 40: D881-886.

36. Tenenhouse HS, Klugerman AH, Neal JL (1989) Effect of phosphonoformic acid, dietary phosphate and the Hyp mutation on kinetically distinct phosphate transport processes in mouse kidney. Biochim Biophys Acta 984: 207-213.

37. Stubbs JR, Liu S, Tang W, Zhou J, Wang Y, et al. (2007) Role of hyperphosphatemia and 1,25-dihydroxyvitamin d in vascular calcification and mortality in fibroblastic growth factor 23 null mice. J Am Soc Nephrol 18: 2116-2124.

38. Ohnishi M, Razzaque MS (2010) Dietary and genetic evidence for phosphate toxicity accelerating mammalian aging. Faseb J 24: 3562-3571.

39. Razzaque MS (2009) Does FGF23 toxicity influence the outcome of chronic kidney disease? Nephrol Dial Transplant 24: 4-7.

40. Ohnishi M, Nakatani T, Lanske B, Razzaque MS (2009) Reversal of mineral ion homeostasis and soft-tissue calcification of klotho knockout mice by deletion of vitamin D 1alpha-hydroxylase. Kidney Int 75: 1166-1172.

41. Dow JA, Romero MF (2010) Drosophila provides rapid modeling of renal development, function, and disease. Am J Physiol Renal Physiol 299: F1237-1244.

42. Steller H (2008) Regulation of apoptosis in Drosophila. Cell Death Differ 15: 1132-1138.

43. Perkins LA, Larsen I, Perrimon N (1992) corkscrew encodes a putative protein tyrosine phosphatase that functions to transduce the terminal signal from the receptor tyrosine kinase torso. Cell 70: 225-236.

44. Chintapalli VR, Wang J, Dow JA (2007) Using FlyAtlas to identify better Drosophila melanogaster models of human disease. Nat Genet 39: 715-720.

45. Mason DA, Goldfarb DS (2009) The nuclear transport machinery as a regulator of Drosophila development. Semin Cell Dev Biol 20: 582-589.

46. Ferrigno P, Posas F, Koepp D, Saito H, Silver PA (1998) Regulated nucleo/cytoplasmic exchange of HOG1 MAPK requires the importin beta homologs NMD5 and XPO1. Embo J 17: 5606-5614.

47. Engel K, Kotlyarov A, Gaestel M (1998) Leptomycin B-sensitive nuclear export of MAPKAP kinase 2 is regulated by phosphorylation. Embo J 17: 3363-3371.

48. Smith RK, Carroll PM, Allard JD, Simon MA (2002) MASK, a large ankyrin repeat and KH domain-containing protein involved in Drosophila receptor tyrosine kinase signaling. Development 129: 71-82.

49. Hou SC, Chan LW, Chou YC, Su CY, Chen X, et al. (2009) Ankrd17, an ubiquitously expressed ankyrin factor, is essential for the vascular integrity during embryogenesis. FEBS Lett 583: 2765-2771.

50. Kilav R, Silver J, Naveh-Many T (1995) Parathyroid hormone gene expression in hypophosphatemic rats. J Clin Invest 96: 327-333.

51. Moallem E, Kilav R, Silver J, Naveh-Many T (1998) RNA-Protein binding and post-transcriptional regulation of parathyroid hormone gene expression by calcium and phosphate. J Biol Chem 273: 5253-5259.

52. Dinur M, Kilav R, Sela-Brown A, Jacquemin-Sablon H, Naveh-Many T (2006) In vitro evidence that upstream of N-ras participates in the regulation of parathyroid hormone messenger ribonucleic acid stability. Mol Endocrinol 20: 1652-1660.

53. Nechama M, Ben-Dov IZ, Briata P, Gherzi R, Naveh-Many T (2008) The mRNA decay promoting factor K-homology splicing regulator protein post-transcriptionally determines parathyroid hormone mRNA levels. Faseb J 22: 3458-3468.

54. Nechama M, Uchida T, Mor Yosef-Levi I, Silver J, Naveh-Many T (2009) The peptidyl-prolyl isomerase Pin1 determines parathyroid hormone mRNA levels and stability in rat models of secondary hyperparathyroidism. J Clin Invest 119: 3102-3114.

55. Jan De Beur S, Finnegan R, Vassiliadis J, Cook B, Barberio D, et al. (2002) Tumors associated with oncogenic osteomalacia express genes important in bone and mineral metabolism. J Bone Miner Res 17: 1102-1110.

56. Martin A, Liu S, David V, Li H, Karydis A, et al. (2011) Bone proteins PHEX and DMP1 regulate fibroblastic growth factor Fgf23 expression in osteocytes through a common pathway involving FGF receptor (FGFR) signaling. Faseb J 25: 2551-2562.

57. Zhao HW, Haddad GG (2011) Review: Hypoxic and oxidative stress resistance in Drosophila melanogaster. Placenta 32 Suppl 2: S104-108.

58. Tower J (2011) Heat shock proteins and Drosophila aging. Exp Gerontol 46: 355-362.

59. Perez VI, Cortez LA, Lew CM, Rodriguez M, Webb CR, et al. (2011) Thioredoxin 1 overexpression extends mainly the earlier part of life span in mice. J Gerontol A Biol Sci Med Sci 66: 1286-1299.

60. Perez VI, Lew CM, Cortez LA, Webb CR, Rodriguez M, et al. (2008) Thioredoxin 2 haploinsufficiency in mice results in impaired mitochondrial function and increased oxidative stress. Free Radic Biol Med 44: 882-892.

61. Tong JJ, Schriner SE, McCleary D, Day BJ, Wallace DC (2007) Life extension through neurofibromin mitochondrial regulation and antioxidant therapy for neurofibromatosis-1 in Drosophila melanogaster. Nat Genet 39: 476-485.

62. Gardner PR, Fridovich I (1991) Superoxide sensitivity of the Escherichia coli aconitase. J Biol Chem 266: 19328-19333.

63. Huang G, Lu H, Hao A, Ng DC, Ponniah S, et al. (2004) GRIM-19, a cell death regulatory protein, is essential for assembly and function of mitochondrial complex I. Mol Cell Biol 24: 8447-8456.

64. Kraus ME, Lis JT (1994) The concentration of B52, an essential splicing factor and regulator of splice site choice in vitro, is critical for Drosophila development. Mol Cell Biol 14: 5360-5370.

65. Lopez-Mejia IC, Vautrot V, De Toledo M, Behm-Ansmant I, Bourgeois CF, et al. (2011) A conserved splicing mechanism of the LMNA gene controls premature aging. Hum Mol Genet 20: 4540-4555.

66. Baldridge D, Shchelochkov O, Kelley B, Lee B (2010) Signaling pathways in human skeletal dysplasias. Annu Rev Genomics Hum Genet 11: 189-217.

67. Schroeder TM, Kahler RA, Li X, Westendorf JJ (2004) Histone deacetylase 3 interacts with runx2 to repress the osteocalcin promoter and regulate osteoblast differentiation. J Biol Chem 279: 41998-42007.

68. Hesse E, Saito H, Kiviranta R, Correa D, Yamana K, et al. (2010) Zfp521 controls bone mass by HDAC3-dependent attenuation of Runx2 activity. J Cell Biol 191: 1271-1283.

69. McGee-Lawrence ME, Bradley EW, Dudakovic A, Carlson SW, Ryan ZC, et al. (2012) Histone deacetylase 3 is required for maintenance of bone mass during aging. Bone.

70. Laplantine E, Rossi F, Sahni M, Basilico C, Cobrinik D (2002) FGF signaling targets the pRb-related p107 and p130 proteins to induce chondrocyte growth arrest. J Cell Biol 158: 741-750.

71. Rossi F, MacLean HE, Yuan W, Francis RO, Semenova E, et al. (2002) p107 and p130 Coordinately regulate proliferation, Cbfa1 expression, and hypertrophic differentiation during endochondral bone development. Dev Biol 247: 271-285.

72. Ito E, Konno Y, Toki T, Terui K (2010) Molecular pathogenesis in Diamond-Blackfan anemia. Int J Hematol 92: 413-418.

73. Gazda HT, Sheen MR, Vlachos A, Choesmel V, O'Donohue MF, et al. (2008) Ribosomal protein L5 and L11 mutations are associated with cleft palate and abnormal thumbs in Diamond-Blackfan anemia patients. Am J Hum Genet 83: 769-780.

74. Ni JQ, Zhou R, Czech B, Liu LP, Holderbaum L, et al. (2011) A genome-scale shRNA resource for transgenic RNAi in Drosophila. Nat Methods 8: 405-407.

75. Dietzl G, Chen D, Schnorrer F, Su KC, Barinova Y, et al. (2007) A genome-wide transgenic RNAi library for conditional gene inactivation in Drosophila. Nature 448: 151-156.

76. Malo N, Hanley JA, Cerquozzi S, Pelletier J, Nadon R (2006) Statistical practice in high-throughput screening data analysis. Nat Biotechnol 24: 167-175.

77. Kaplow IM, Singh R, Friedman A, Bakal C, Perrimon N, et al. (2009) RNAiCut: automated detection of significant genes from functional genomic screens. Nat Methods 6: 476-477.

78. Jackson AL, Bartz SR, Schelter J, Kobayashi SV, Burchard J, et al. (2003) Expression profiling reveals off-target gene regulation by RNAi. Nat Biotechnol 21: 635-637.

79. Kulkarni MM, Booker M, Silver SJ, Friedman A, Hong P, et al. (2006) Evidence of off-target effects associated with long dsRNAs in Drosophila melanogaster cell-based assays. Nat Methods 3: 833-838.
